# Spin-regulated Fe-N-C catalyst enabled by adjusting coordination nitrogen species for robust oxygen reduction

**DOI:** 10.1093/nsr/nwaf061

**Published:** 2025-02-20

**Authors:** Ning Wang, Chao Meng, Bin Wang, Xiaojie Tan, Yi Wan, Yang Yang, Deyu Kong, Wanli Wang, Fengliang Cao, Alistair J Fielding, Lina Li, Mingbo Wu, Han Hu

**Affiliations:** State Key Laboratory of Heavy Oil Processing, Institute of New Energy, College of Chemistry and Chemical Engineering, China University of Petroleum (East China), Qingdao 266580, China; State Key Laboratory of Heavy Oil Processing, Institute of New Energy, College of Chemistry and Chemical Engineering, China University of Petroleum (East China), Qingdao 266580, China; State Key Laboratory of Heavy Oil Processing, Institute of New Energy, College of Chemistry and Chemical Engineering, China University of Petroleum (East China), Qingdao 266580, China; State Key Laboratory of Heavy Oil Processing, Institute of New Energy, College of Chemistry and Chemical Engineering, China University of Petroleum (East China), Qingdao 266580, China; State Key Laboratory of Heavy Oil Processing, Institute of New Energy, College of Chemistry and Chemical Engineering, China University of Petroleum (East China), Qingdao 266580, China; State Key Laboratory of Heavy Oil Processing, Institute of New Energy, College of Chemistry and Chemical Engineering, China University of Petroleum (East China), Qingdao 266580, China; State Key Laboratory of Heavy Oil Processing, Institute of New Energy, College of Chemistry and Chemical Engineering, China University of Petroleum (East China), Qingdao 266580, China; State Key Laboratory of Heavy Oil Processing, Institute of New Energy, College of Chemistry and Chemical Engineering, China University of Petroleum (East China), Qingdao 266580, China; State Key Laboratory of Heavy Oil Processing, Institute of New Energy, College of Chemistry and Chemical Engineering, China University of Petroleum (East China), Qingdao 266580, China; Centre for Natural Products Discovery, School of Pharmacy and Biomolecular Sciences, Liverpool John Moores University, Liverpool L3 3AF, UK; Shanghai Synchrotron Radiation Facility (SSRF), Zhangjiang Lab, Shanghai Institute of Applied Physics, Shanghai Advanced Research Institute, Chinese Academy of Sciences, Shanghai 201204, China; State Key Laboratory of Heavy Oil Processing, Institute of New Energy, College of Chemistry and Chemical Engineering, China University of Petroleum (East China), Qingdao 266580, China; State Key Laboratory of Heavy Oil Processing, Institute of New Energy, College of Chemistry and Chemical Engineering, China University of Petroleum (East China), Qingdao 266580, China

**Keywords:** oxygen reduction reaction, Fe-N-C, graphitic nitrogen, spin state, Zn-air battery

## Abstract

Fe-N-C catalysts have emerged as a promising substitute for the expensive Pt/C to boost the oxygen reduction reaction (ORR). However, conventional Fe-N_4_ active sites, which generally feature a low-spin configuration, strongly adsorb oxygen intermediates and necessitate structural optimization of the active sites for improved performance. Herein, graphitic nitrogen (N_GC_) adjacent to the Fe-N_4_ centers is straightforwardly introduced to modulate the spin state of Fe-N-C catalysts after elucidating the influence of nitrogen species on the Fe-N_4_ sites. Theoretical calculations demonstrate that the adjacent N_GC_ can effectively regulate the spin state of the active Fe sites, which enables electron filling from Fe to the anti-bonding π* orbital of oxygen species and optimizes the *OH desorption for accelerated ORR. Inspired by this, such catalysts are cost-effectively prepared by a rational combination of electrospinning and controlled thermal annealing using inexpensive precursors. The optimal catalyst shows superior ORR activity to the benchmark Pt/C, and excellent durability, with a minor voltage decay of 11 mV after 10 000 cycles. The spin-state-promoted performance enhancement is confirmed by a series of *in-situ* characterizations. The remarkable performance of the optimized catalyst is further confirmed in Zn-air batteries (ZABs) with a peak power density of 225 mW cm^−2^. Moreover, quasi-solid ZABs using this catalyst realize excellent performance even under bending conditions and successfully power electronic devices, including a mobile phone and an electronic watch. This work correlates the spin state of catalysts and oxygen reduction performance, providing an alternative strategy for regulating the performance of electrocatalysts as well as promoting their application in wearable electronics.

## Teaser

This study establishes a correlation between the ORR activity of Fe-N-C catalysts and their spin state, proposing a straightforward approach for spin regulation by adjusting coordination graphitic nitrogen.

## INTRODUCTION

Global efforts to address the challenges of climate change and the energy crisis have expedited the development of electrochemical energy storage and conversion technologies [1–5]. The oxygen reduction reaction (ORR), a key process for many energy-related electrochemical technologies, for example Zn-air batteries (ZABs), has thus aroused tremendous interest in the past decade [6–9]. However, the ORR entails multi-electron-proton transfer with sluggish kinetics, thus demanding efficient electrocatalysts to accelerate the ORR [10]. Although commercial Pt/C shows high ORR activity, the scarcity and high cost of Pt largely hinders its widespread application [11,12]. Consequently, developing alternatives to Pt/C with non-precious metals for accelerated ORR kinetics is urgently required.

Recently, emerging transition metal-nitrogen-carbon (M-N-C) catalysts, in which the active metal is typically coordinated by four pyridinic N sites, especially the Fe-N-C variant, have stood out as promising candidates to replace the commercial Pt/C catalyst for enhanced ORR [13–15]. The bond strength between the oxygen-containing intermediates and the active Fe sites, a crucial factor that determines ORR performance, is largely dependent on the electron occupancy at the 3*d* orbital of Fe, alternatively the spin state [16–18]. The typical Fe-N_4_ configurations of Fe-N-C catalysts exhibit an empty anti-bonding orbital because of the large electronegativity of the coordinated N species. Consequently, the Fe in such a Fe-N-C configuration is generally at a low spin state (LS, t_2g_5e_g_0), which strongly adsorbs the oxygen intermediates and impedes the subsequent steps of ORR [19]. Recent research has suggested that an effective approach to addressing the strong adsorption issue is to modulate the low spin state of active Fe sites, which can be achieved by introducing foreign species adjacent to the Fe-N_4_ centers. The foreign species surrounding the coordinated single atomic metal centers can regulate the distribution of electrons in the 3*d* orbital of the metal, modulating the adsorption/desorption behavior of the active metals due to their adjusted spin states [20,21]. Wang's team implanted Fe clusters around the Fe-N_4_ centers to introduce electrons into the *d*_z_^2^ orbital of atomically dispersed Fe, contributing to boosted ORR activity [19]. Zhang's research group introduced Mn single atoms adjacent to Fe to form a dual-metal atomically dispersed configuration and observed an essentially facilitated adsorption of the oxygen species at the active Fe sites [22]. Zhai proposed that S doping can effectively regulate the spin state of atomically dispersed Fe for tunable ORR activity [23]. Despite these advances, straightforward and cost-effective methods for spin regulation are still urgently needed [19,24]. Typical syntheses of Fe-N-C catalysts involve thermal annealing of the mixture containing metal salts and organic precursors as nitrogen and carbon sources [25–27]. The nitrogen content generally overwhelms the required amount to coordinate Fe, and the as-obtained catalysts, in fact, contain quite some nitrogen species such as pyrrolic nitrogen and pyridinic nitrogen in the surroundings of the Fe-N_4_ centers. The nitrogen species around Fe-N_4_ potentially have the ability to regulate the spin configuration of the active centers, and a clear understanding of the relationship between them could contribute to an effective approach to spin regulation of the Fe-N_4_ centers. Nevertheless, the actual mechanism still remains elusive.

Herein, we systematically investigated the influence of nitrogen species on the spin state and ORR activity of Fe-N_4_ centers, with adjacent graphitic nitrogen (N_GC_) being the most favorable in terms of regulating ORR activity. Specifically, the N_GC_ can induce electron delocalization in the 3*d* orbital of Fe sites to optimize the adsorption and desorption of oxygen intermediates. To deliberately implant N_GC_ surrounding the Fe-N_4_ centers, the Fe-N_4_-C catalysts with a high nitrogen content are annealed at a higher temperature whereupon part of other nitrogen species are directionally converted into N_GC_. Systematic structure analysis proposes the successful production of the Fe-N_4_-C with adjacent N_GC_ (denoted as Fe-N_4_/N_GC_-C). Moreover, electron paramagnetic resonance (EPR) spectroscopy combined with magnetic characterization confirms a spin transition from a low to high state after the introduction of N_GC_. Consequently, the Fe-N_4_/N_GC_-C catalyst demonstrates superior ORR performance and significant durability (voltage attenuation of only 11 mV after 10 000 cycles) compared with the commercial Pt/C. Then, *in-situ* electrochemical impedance spectroscopy (EIS), *in-situ* attenuated total reflectance surface-enhanced infrared absorption spectroscopy (ATR-SEIRAS) and *in-situ* Raman were employed to monitor the ORR process, in which the facilitated O_2_ adsorption and *OH desorption are observed. Moreover, the Fe-N_4_/N_GC_-C was used as the cathode catalyst for ZABs using liquid and quasi-solid electrolytes, separately, which can deliver an unexceptionable high peak density of 225 and 136 mW cm^−2^, respectively. Quasi-solid counterparts with the Fe-N_4_/N_GC_-C catalyst can even work smoothly at bent conditions and power electronic devices including mobile phones and electronic watches.

## RESULTS AND DISCUSSION

### Theoretical calculation

**Figure 1.**
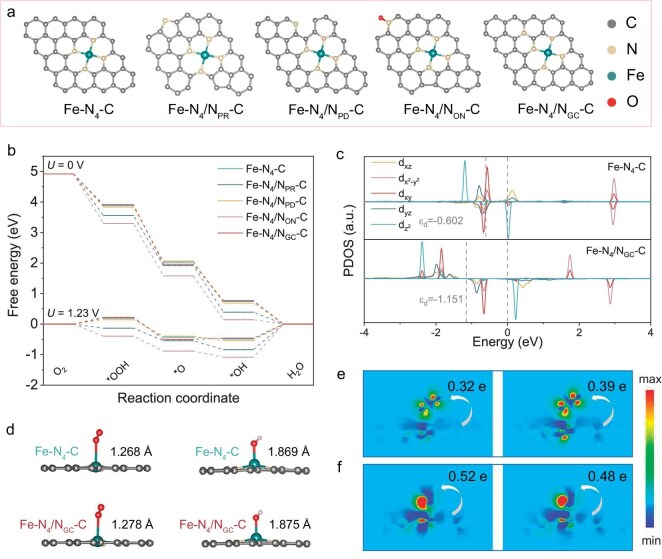
(a) Structure models of Fe-N_4_-C with different adjacent N species including Fe-N_4_/N_PR_-C, Fe-N_4_/N_PD_-C, Fe-N_4_/N_ON_-C and Fe-N_4_/N_GC_-C. (b) Gibbs free energy diagrams of ORR on different structures at 0 V and 1.23 V. (c) PDOS of the 3*d* orbitals analysis at the Fe site for Fe-N_4_-C and Fe-N_4_/N_GC_-C. (d) The length variation of the O-O bond and Fe-O bond with O_2_ and *OH adsorbed on Fe-N_4_-C and Fe-N_4_/N_GC_-C. The Bader charges transferred from Fe-N_4_-C and Fe-N_4_/N_GC_-C to (e) O_2_ and (f) *OH.

To explore the impact of different adjacent nitrogen species on the ORR performance of the Fe-N_4_-C, density functional theory (DFT) calculations were conducted on Fe-N_4_-C and a series of structures with different nitrogen species implanted adjacent to the Fe-N_4_ centers, including pyrrolic N (denoted as Fe-N_4_/N_PR_-C), pyridinic N (Fe-N_4_/N_PD_-C), oxidized N (Fe-N_4_/N_ON_-C) and graphitic N (Fe-N_4_/N_GC_-C) (Fig. 1a). The energy variation of the ORR was evaluated after the intermediates were adsorbed on different models (Figs S1–S5 in the online supplementary data). Figure 1b and Tables S1 and S2 illustrate the Gibbs free energy of oxygen-containing-species adsorption on the aforementioned structures at different potentials. With the potential *U* = 0 V, all catalysts show a downward trend in the reaction path, indicating the thermodynamically favorable feature. When the potential increases to 1.23 V, the final step of (*OH + e^–^ → OH^–^ + *) is rate determining, in which the desorption energy of Fe-N_4_-C is 0.49 eV for the release of *OH. Compared with other catalysts, the desorption of *OH in Fe-N_4_/N_GC_-C is optimized to 0.46 V, which facilitates the ORR kinetics. To further demonstrate the effect of N_GC_ on ORR performance, Fe-N_4_/N_GC_-C structures with N_GC_ at different sites were built (Fig. S6). As shown in Fig. S7, all the Fe-N_4_/N_GC_-C catalysts show a lower barrier for ORR than Fe-N_4_-C, which confirms that the presence of N_GC_ has a significant effect on the catalytic performance of Fe-N_4_-C.

According to molecular orbital theory, partially vacant 3*d* orbitals of metal sites are necessary to form bonds with O_2_ [20,28]. The projected density of states (PDOS) of Fe 3*d* orbitals in the Fe-N_4_-C and Fe-N_4_/N_GC_-C models (Fig. 1c) was thus compared, both of which exhibit spin-up and spin-down densities. Specifically, Fe-N_4_/N_GC_-C shows a larger spin-polarized region, indicating a higher occupancy of spin-polarized electrons in the 3*d* orbitals. These spin-polarized electrons help bind to two unpaired π* electrons in the triplet O_2_ with the same spin orientation, thereby enhancing the adsorption of O_2_ at the initial step of the ORR [29]. In addition, the *d*-band center of Fe-N_4_/N_GC_-C (−1.151 eV) is shifted downward compared to that of the Fe-N_4_-C (−0.602 eV), which means that the Fe-N_4_/N_GC_-C catalyst can efficiently desorb the subsequent oxygen-containing intermediates to promote the continuity of the ORR [30].

To intuitively understand the effect of N_GC_ on ORR activity, we calculated the differential charge density and Bader charge of different models. As shown in Fig. 1d and e, the charge density difference between Fe and O_2_ indicates that more electrons are transferred from Fe-N_4_/N_GC_-C (0.39 e) to O_2_ compared to Fe-N_4_-C (0.32 e), enabling a longer O-O bond on Fe-N_4_/N_GC_-C. In contrast, the electron transfer from the catalyst to *OH is reduced from 0.52 e for Fe-N_4_-C to 0.48 e for Fe-N_4_/N_GC_-C, suggesting a weaker adsorption of *OH on Fe-N_4_/N_GC_-C (Fig. 1f). Meanwhile, the Fe-O bond between the adsorbed *OH intermediate and Fe-N_4_/N_GC_-C is longer than that in the Fe-N_4_-C catalyst (Fig. 1d). This facilitates the rapid dissociation of the Fe-OH bond for re-exposing the active sites, essentially promoting the 4-electron reaction process.

### Synthesis and characterization

Compared with other nitrogen species, N_GC_ is more thermally stable. Therefore, by simply annealing the as-prepared Fe-N_4_-C catalysts at a higher temperature, part of the uncoordinated nitrogen will be converted into the thermally stable N_GC_ near the catalytic active center [31]. Alternatively, the thermally unstable nitrogen species would be released to react with the carbon matrix, contributing to N_GC_ [32]. To synthesize such a structure, a precursor solution containing Fe doped zeolitic imidazolate framework-8 (Fe-ZIF-8) (Fig. S8), polyacrylonitrile (PAN) and polyvinyl pyrrolidone (PVP) was firstly electrospun to produce self-supported nanofibers, which were then stabilized and carbonized at 800°C to form Fe-N_4_-C. Following this, an additional annealing step at a higher temperature was conducted to deliberately introduce N_GC_. Compared to other strategies for regulating active centers, this process, which uses inexpensive precursors (Table S3) and a simple synthesis method, is more straightforward and cost effective.

**Figure 2.**
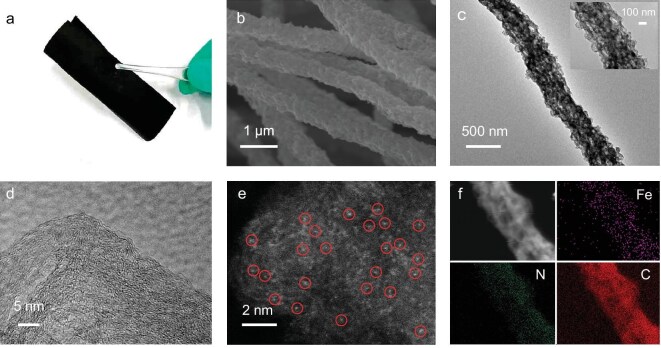
(a) Digital image of Fe-N_4_/N_GC_-C. (b) SEM, (c) TEM, (d) HR-TEM, (e) AC HAADF-STEM, and (f) EDS elemental mapping images of Fe-N_4_/N_GC_-C.

The morphology of Fe-N_4_-C and structures obtained at higher temperatures are compared in Fig. S9. All these structures show interconnected networks made of uniform nanofibers. Nevertheless, the fibers begin to fracture at the annealing temperature of 1100°C (Fig. S9i and j). To highlight the impact of N_GC_ and reduce the influence of other factors, such as residual Zn (Fig. S10), the analysis is then mainly focused on the structure obtained at 1000°C [33]. The as-obtained Fe-N_4_/N_GC_-C at 1000°C shows remarkable flexibility, maintaining an intact sheet structure even under bending stress (Fig. 2a and Video S1). In addition, carbon nanofibers (CNFs) as a reference structure was also prepared using an identical method to Fe-N_4_/N_GC_-C except for the absence of Fe in the precursor. The scanning electron microscopy (SEM) images of CNFs (Fig. S9a and b), Fe-N_4_-C (Fig. S9c and d) and Fe-N_4_/N_GC_-C (Fig. 2b, and S9g and h) at higher magnification reveal rough surfaces that may facilitate the exposure of active sites. The micropores inherited from ZIF, as well as the mesopores left by PVP pyrolysis and Zn evaporation together constitute the hierarchical porous characteristics of Fe-N_4_/N_GC_-C, as depicted in the transmission electron microscopy (TEM) images (Fig. 2c and Fig. S11) [34]. The X-ray diffraction (XRD) patterns show only a broad diffraction peak for Fe-N_4_-C and Fe-N_4_/N_GC_-C, confirming their amorphous nature (Fig. S12). The content of Fe was determined by inductively coupled plasma optical emission spectroscopy (ICP-OES) as 0.52 wt% and 0.54 wt% for Fe-N_4_-C and Fe-N_4_/N_GC_-C, respectively. The metal species are uniformly distributed without aggregations from high-resolution transmission electron microscopy (HR-TEM) (Fig. 2d), indicating atomically dispersed Fe species. Atomic-column high-angle annular dark-field scanning transmission electron microscopy (AC HAADF-STEM) analysis of the Fe-N_4_/N_GC_-C structure (Fig. 2e) displays isolated bright spots, highlighted by the red circles, representing the atomically dispersed Fe. Energy dispersive X-ray spectrometer (EDS) elemental mapping reveals a uniform distribution of Fe, C and N in the whole carbon skeleton (Fig. 2f).

**Figure 3.**
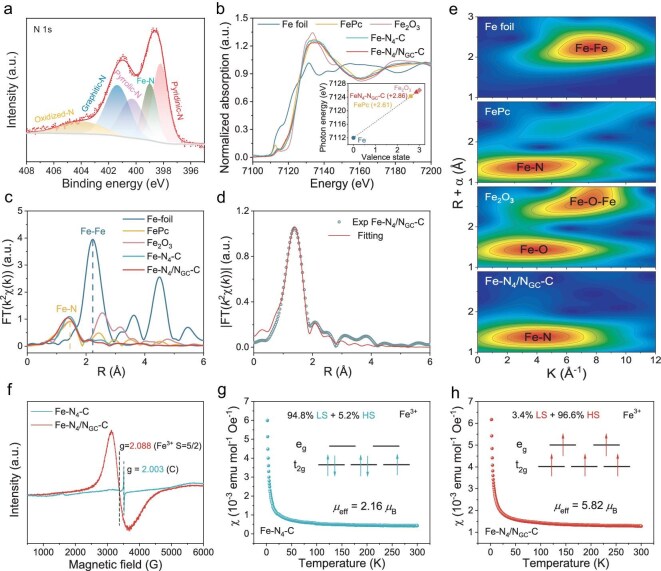
(a) N 1*s* XPS spectra of Fe-N_4_/N_GC_-C. (b) XANES of the Fe K-edge, and (c) FT-EXAFS in the R space of Fe-N_4_/N_GC_-C and reference samples. (d) FT-EXAFS fitting curve at the Fe K-edge of Fe-N_4_/N_GC_-C. (e) Wavelet transforms for the k^2^-weighted Fe K-edge EXAFS signals for Fe-N_4_/N_GC_-C and reference samples. (f) EPR spectra of the Fe-N_4_-C and Fe-N_4_/N_GC_-C. Magnetic susceptibility of (g) Fe-N_4_-C and (h) Fe-N_4_/N_GC_-C.

The chemical state of the catalysts was then analyzed using different spectral technologies. As compared to the X-ray photoelectron spectroscopy (XPS) of Fe-N_4_-C and Fe-N_4_/N_GC_-C, a much higher content of N_GC_ is observed for Fe-N_4_/N_GC_-C (Fig. 3a, Fig. S14, and Table S4). In addition to the nitrogen species coordinated with Fe, the N_GC_ represents the dominant N species for the Fe-N_4_/N_GC_-C. After a second thermal annealing at a higher temperature, the Fe-N_4_/N_GC_-C exhibits a better thermal stability (Fig. S15) and higher graphitization degree (Fig. S16), which could result in improved electron transfer and durability for the catalyst. To further explore the valence state and coordination structure of Fe in Fe-N_4_-C and Fe-N_4_/N_GC_-C, the K-edge X-ray absorption spectroscopy (XAS) of Fe was measured using fluorescence mode. The valence state of Fe in Fe-N_4_-C and Fe-N_4_/N_GC_-C was determined through normalized Fe K-edge X-ray absorption near edge spectroscopy (XANES) data (Fig. 3b). The Fe K-edge XANES spectra of Fe-N_4_-C and Fe-N_4_/N_GC_-C are situated between those of the Fe foil and Fe_2_O_3_ standard sample, closely resembling that of FePc, which indicates the presence of cationic Fe states in the samples [35]. The valence state of Fe was estimated from the derivative spectrum of XANES, as shown in the inset of Fig. 3b. The average valence state of Fe in Fe-N_4_/N_GC_-C is predominantly between +2 and +3, and close to +3. The Fourier transform (FT) k^2^-weighted extended X-ray absorption fine structure (EXAFS) spectra of the catalysts were further analyzed (Fig. 3c). The Fe-N_4_/N_GC_-C catalyst shows a prominent peak associated with the Fe-N first coordination shell at ∼1.4 Å. No Fe-Fe coordination peak was detected, indicating that Fe is atomically dispersed, consistent with the results of the AC HAADF-STEM analysis. Fitting the EXAFS curves permits the quantitative analysis of structural parameters around the Fe centers (Fig. 3d, Fig. S17, and Table S5), and a coordination number of 4 is observed for both Fe-N in Fe-N_4_-C and Fe-N_4_/N_GC_-C. The EXAFS wavelet transform (WT) spectra show contour lines similar to those of FePc, further confirming the existence of the Fe-N coordination pathway in Fe-N_4_-C and Fe-N_4_/N_GC_-C (Fig. 3e and Fig. S18).

Numerous studies indicate that the electrocatalytic performance of Fe-N_4_-C catalysts is closely related to the 3*d* electron configuration of Fe [19,36]. To elucidate the electron configuration of the 3*d* orbitals in Fe, EPR and magnetic susceptibility were employed. An EPR signal corresponding to the high-spin state of Fe^3+^ (HS, S = 5/2) appears at g = 2.088 (Fig. 3f) [37,38]. Compared with Fe-N_4_-C, the mass-normalized EPR signal intensity of Fe-N_4_/N_GC_-C is much higher, indicating that the N_GC_ triggers the spin state transition. In addition, the EPR spectrum of CNFs only shows the carbon signal at g = 2.003 (Fig. S22). The EPR findings align well with the effective magnetic moment (*μ*_eff_) calculated based on Langevin theory, with values of 5.82 *µ*_B_ and 2.16 *µ*_B_ for Fe-N_4_/N_GC_-C and Fe-N_4_-C (Fig. 3g and h), respectively. The corresponding number of unpaired electrons (*n*) is 4.9 for Fe-N_4_/N_GC_-C and 1.4 for Fe-N_4_-C, according to the equation *μ*_eff_^2^ = *n*(*n* + 2)*µ*_B_^2^, further indicating a high-spin configuration of Fe in Fe-N_4_/N_GC_-C. High-spin Fe sites possess more unpaired electrons in *e*_g_ orbitals, which facilitates penetration into the anti-bonding π* orbital of O_2_. Additionally, the spin-orbit coupling effect between the high-spin Fe site and O_2_ promotes the formation of the Fe-O bond, thereby enhancing O_2_ adsorption [22].

### Electrochemical ORR performance

**Figure 4.**
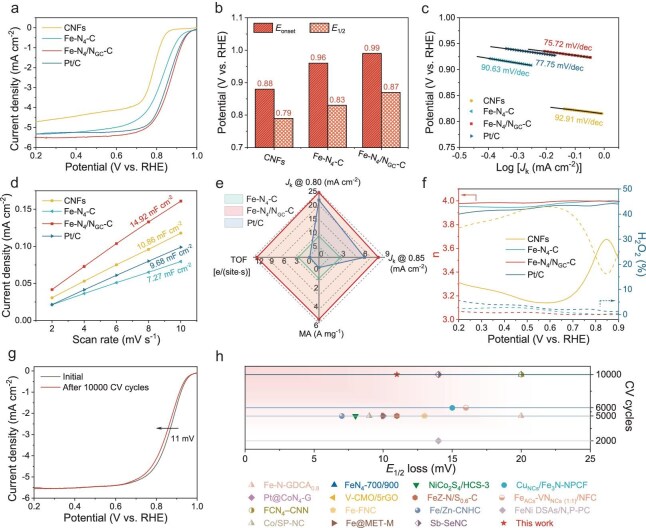
ORR activity of the Fe-N_4_/N_GC_-C catalyst in O_2_-saturated 0.1 M KOH solution. (a) LSV curves, (b) *E*_onset_ and *E*_1/2_ vs. RHE, (c) the corresponding Tafel plots, and (d) the variation of current densities at different scan rates for Fe-N_4_/N_GC_-C and reference samples. (e) Comparisons of *J*_k_ (at 0.80 V and 0.85 V), TOF and MA of Fe-N_4_/N_GC_-C, Fe-N_4_-C and Pt/C. (f) The number of electron transfers (n) and the yield of H_2_O_2_ for the related samples. (g) ORR polarization LSV curves of Fe-N_4_/N_GC_-C before and after 10 000 potential cycles at a scan rate of 100 mV s^−1^. (h) Comparison of *E*_1/2_ loss of recently reported catalysts in O_2_-saturated 0.1 M KOH solution (Table S7).

To evaluate the ORR activity of the catalyst with high spin state, cyclic voltammetry (CV) and linear sweep voltammetry (LSV) curves of Fe-N_4_/N_GC_-C were compared with those of Fe-N_4_-C, Pt/C and CNFs in O_2_-saturated 0.1 M KOH solution. The CV curves (Fig. S24) show that the Fe-N_4_/N_GC_-C catalyst exhibits a higher oxygen reduction peak than other catalysts, confirming its higher ORR activity. Meanwhile, the LSV results (Fig. 4a and b, and Fig. S25) reveal that Fe-N_4_/N_GC_-C displays an exceptional initial potential (*E*_onset_) of 0.99 V vs. reversible hydrogen electrode (RHE) and a half-wave potential (*E*_1/2_) of 0.87 V vs. RHE. The Tafel slope (Fig. 4c) of Fe-N_4_/N_GC_-C (75.72 mV dec^−1^) is smaller than that of CNFs (92.91 mV dec^−1^), Fe-N_4_-C (90.63 mV dec^−1^) and Pt/C (77.75 mV dec^−1^). The electrochemical surface area (ECSA) of the catalyst was determined through its linear correlation with electrochemical double-layer capacitance (*C*_dl_). The *C*_dl_ value (Fig. 4d and Fig. S26) for Fe-N_4_/N_GC_-C (14.92 mF cm^−2^) surpasses that of CNFs (10.86 mF cm^−2^), Fe-N_4_-C (7.27 mF cm^−2^) and Pt/C (9.68 mF cm^−2^). As shown in Fig. 4e and Table S6, Fe-N_4_/N_GC_-C demonstrates the highest kinetic current density (*J*_k_) at 0.80 and 0.85 V vs. RHE, with values of 24.76 and 8.13 mA cm^−2^, respectively, superior to Fe-N_4_-C (8.66 mA cm^−2^, 2.91 mA cm^−2^) and Pt/C (21.90 mA cm^−2^, 6.25 mA cm^−2^). Notably, Fe-N_4_/N_GC_-C exhibits higher mass activity (MA) and turnover frequency (TOF) than other catalysts, confirming its excellent ORR performance. In conjunction with the structural analysis, the variation trend of the activity among these catalysts indicates that ORR performance, in our case, is mainly dependent on the Fe-N_4_-C centers, instead of other structural features, such as the residual Zn.

The ORR pathway of Fe-N_4_/N_GC_-C was evaluated by conducting LSV curves at various rotating speeds (Fig. S27). The Koutecky-Levich (K-L) plot indicates a well-defined linear relationship in the limiting current density (*J*_L_) region, confirming first-order reaction kinetics in relation to the concentration of dissolved oxygen [39]. The *E*_onset_ remains unchanged at different rotating speeds, while the *J*_L_ gradually increases with an elevation of rotating speed due to a reduction in concentration polarization at higher rotating speeds [29]. The average electron transfer number (*n*) of Fe-N_4_/N_GC_-C, calculated using the K-L equation at different potentials, is 3.99, close to the theoretical value of 4 for the direct 4 e^–^ reaction pathway. The *n* and H_2_O_2_ yields were further evaluated through the rotating ring disk electrode (Fig. 4f), yielding results consistent with the K-L results. In the potential range of 0.2–0.9 V vs. RHE, Fe-N_4_/N_GC_-C achieves an H_2_O_2_ yield below 2%, demonstrating high selectivity towards OH⁻ in the ORR reaction, which may ensure long-term stability for use as the cathode catalyst of ZABs [40]. The durability of Fe-N_4_/N_GC_-C was also assessed. After 10 000 accelerated CV cycles, the *E*_1/2_ of Fe-N_4_/N_GC_-C only exhibits a negligible decay of 11 mV (Fig. 4g), which surpasses most of the previously reported catalysts under similar conditions (Fig. 4h and Table S7). Such excellent stability could be attributed to improved structural stability after high-temperature annealing.

### Mechanism of electrocatalytic ORR

**Figure 5.**
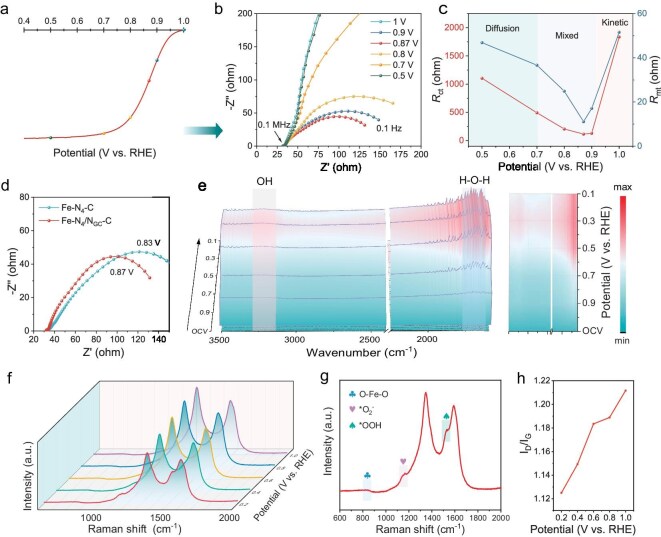
Mechanism analysis of the improved ORR activity of the Fe-N_4_/N_GC_-C. (a) The LSV curve of the Fe-N_4_/N_GC_-C at 1600 r/min in O_2_-saturated 0.1 M KOH solution and (b) the related Nyquist plots of Fe-N_4_/N_GC_-C at different potentials. (c) The *R*_ct_ and *R*_mt_ at different potentials for Fe-N_4_/N_GC_-C. (d) Comparison of Nyquist plots between Fe-N_4_-C and Fe-N_4_/N_GC_-C at *E*_1/2_ vs. RHE. (e) *In-situ* ATR-SEIRAS spectra and (f) *in-situ* Raman spectra of Fe-N_4_/N_GC_-C. (g) Raman spectrum of Fe-N_4_/N_GC_-C tested at 0.2 V vs. RHE. (h) The corresponding I*_D_*/I*_G_* values of Fe-N_4_/N_GC_-C at different potentials.

To gain insight into the enhanced ORR activity of Fe-N_4_/N_GC_-C, a series of *in-situ* characterizations, including EIS, ATR-SEIRAS and Raman measurements, were conducted in a 0.1 M KOH solution saturated with O_2_. EIS tests were conducted at different potentials in the frequency range of 0.1 Hz to 0.1 MHz (Fig. 5a and Fig. S31a), and the Nyquist plots were then fitted into an equivalent circuit model (Fig. S32). The fitting parameters are shown in Tables S8 and S9. Specifically, *R*_s_ represents the solution resistance, while *R*_ct_ and *R*_mt_ correspond to the charge transfer resistance and diffusion resistance, respectively. The correlation between the applied potential and *R*_ct_, as well as *R*_mt_, can reveal the kinetics characteristics of oxygen-related species evolution [41,42]. As shown in Fig. 5b and c, in the dynamic control region (1 V vs. RHE), both *R*_ct_ and *R*_mt_ values are high due to the weak charge transfer between the electrode and the reaction interface. As the potential decreases, the *R*_ct_ drops significantly. When the applied potential reaches *E*_1/2_, both *R*_ct_ and *R*_mt_ attain their minimum values, indicating that direct oxygen reduction occurs on the electrode surface, agreeing well with the experimental observations [43]. When the applied potential falls below *E*_1/2_, diffusion control becomes dominant, leading to a gradual increase in *R*_ct_ and *R*_mt_ because of H_2_O formation and desorption at the electrode surface. Compared with the Fe-N_4_-C catalyst (Fig. 5d and Fig. S31b), the Fe-N_4_/N_GC_-C exhibits a smaller arc radius at *E*_1/2_, meaning higher electronic transfer efficiency. This behavior can be attributed to the facilitated electron transfer through the spin channel enabled by the high-spin Fe, thereby enhancing the catalytic activity of Fe-N_4_/N_GC_-C [44]. At high potentials, the disparity in *R*_ct_ values between Fe-N_4_-C and Fe-N_4_/N_GC_-C further underscores the optimization of the coordination environment at the Fe sites in Fe-N_4_/N_GC_-C, improving O₂ adsorption, accelerating intermediate formation, and enhancing *OH desorption, consistent with DFT calculations [44,45]. *In-situ* ATR-SEIRAS and *in-situ* Raman measurements were also carried out to elucidate key intermediates and structural evolution. As depicted in Fig. 5e, the absorbance intensity at 3220 cm⁻¹ during ATR-SEIRAS measurements positively correlates with the applied overpotential, corresponding to the stretching vibration mode of the adsorbed OH species. The weaker absorption signals suggest that the active sites facilitate rapid desorption after the *OH adsorption, enhancing the kinetics of the 4-electron transfer process. Additionally, a peak related to the bending vibrational mode of OH within H_2_O molecules, observed at 1680 cm⁻¹, may be associated with the adsorption of H₂O during the ORR [46]. *In-situ* Raman spectra provide the bonding information of oxygen-containing intermediates (Fig. 5f and g). The peak at 827 cm^−1^ is ascribed to the asymmetric stretching mode of oxygen in the O-Fe-O bond [47]. As the overpotential decreases to 0.2 V vs. RHE, two additional peaks emerge at 1150 and 1530 cm^−1^, attributed to the O-O stretching vibrations of O_2_^–^ and *OOH, respectively [48]. The intensities of these peaks increased with the rising overpotential, indicating a strong interaction between the active sites and both O_2_^−^/*OOH. As shown in Fig. 5h, the ratios of I*_D_*/I*_G_* for Fe-N_4_/N_GC_-C decrease with increasing overpotential, suggesting dislocation within the carbon matrix [46]. These findings offer valuable insights into the ORR mechanism for Fe-N-C electrocatalysts.

### Zn-air batteries

**Figure 6.**
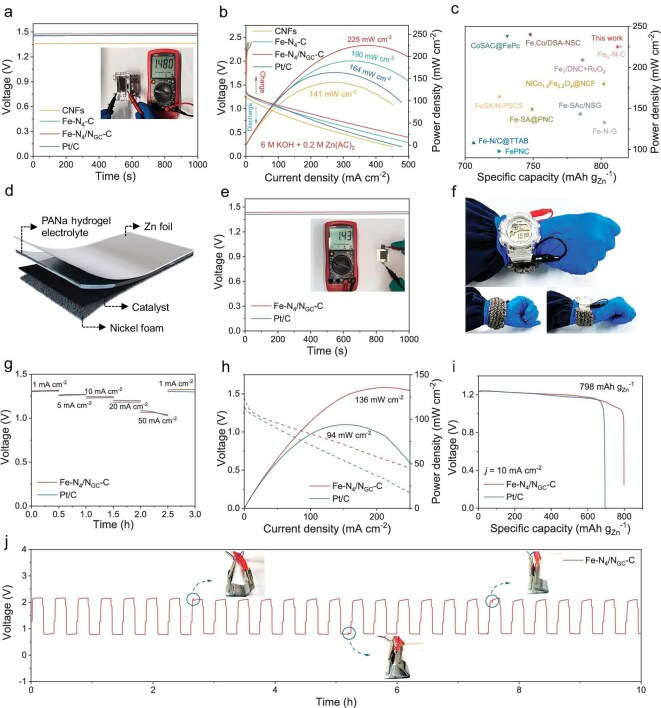
Electrochemical performance of Fe-N_4_/N_GC_-C in ZABs. (a) Open circuit potentials. (b) Charge and discharge polarization plots of the ZABs with Fe-N_4_/N_GC_-C as the catalyst in an aqueous electrolyte with 6 M KOH and 0.2 M Zn(CH_3_COO)_2_. (c) Comparison of the specific capacity and power density of the ZABs using Fe-N_4_/N_GC_-C catalysts and other catalysts listed in Table S10. (d) Structural illustration of a quasi-solid ZAB using a sodium polyacrylate (PANa) hydrogel electrolyte. (e) Open circuit potential of the quasi-solid ZABs with different catalysts. (f) Photograph of an electronic watch powered by two series-connected quasi-solid ZABs. (g) Current density discharge plots, (h) discharge polarization plots, and (i) specific capacity plots of the quasi-solid ZABs with Fe-N_4_/N_GC_-C and Pt/C as the catalysts. (j) Long-term charge and discharge profiles at 10 mA cm^–2^ under different bending angles for the quasi-solid ZABs with Fe-N_4_/N_GC_-C as the catalyst.

To demonstrate the practical performance of Fe-N_4_/N_GC_-C, ZABs with Fe-N_4_/N_GC_-C as the cathode catalyst were assembled using both liquid and quasi-solid electrolytes (Fig. S34a and Fig. 6d). The ZAB using a liquid electrolyte with the Fe-N_4_/N_GC_-C catalyst has a high open circuit voltage (OCV) of 1.48 V (Fig. 6a), capable of powering an electronic timer for several hours (Fig. S34c). It achieves a high peak power density of 225 mW cm^−2^ (Fig. 6b) and specific capacity of 812 mAh g_Zn_^−1^ at 10 mA cm^−2^ (Fig. S34d), outperforming the ZABs employing CNFs (141 mW cm^−2^ and 602 mAh g_Zn_^−1^), Fe-N_4_-C (190 mW cm^−2^ and 782 mAh g_Zn_^−1^), 20% Pt/C catalysts (164 mW cm^−2^ and 733 mAh g_Zn_^−1^), and most of the recently reported counterparts using Fe-based ORR electrocatalysts (Fig. 6c and Table S10). Additionally, this ZAB demonstrates superior charge-discharge cycle stability to the one employing a Pt/C catalyst (Fig. S34e), with a smaller potential gap and sustained high stability over a continuous charge-discharge test of at least 1200 cycles.

As for the ZABs employing a quasi-liquid electrolyte (Fig. 6d), the employment of Fe-N_4_/N_GC_-C and Pt/C as the cathode catalysts contribute to an OCV of 1.43 V and 1.41 V, respectively (Fig. 6e). Flexible quasi-solid ZABs using Fe-N_4_/N_GC_-C cathode catalysts connected in series can power an electronic watch and even a mobile phone (Fig. 6f and Fig. S35, and Video S2). Along with the excellent rate capability of the ZABs (Fig. 6g and Table S11), their series connection could potentially serve as a powerful charger. Moreover, the batteries, which mainly consist of biosafe components, permit them to be power candidates for wearable electronics [7]. The power density and specific capacity for the quasi-solid ZAB using Fe-N_4_/N_GC_-C is much better than its counterpart with the Pt/C catalyst (94 mW cm^−2^, 690 mAh g_Zn_^−1^) (Fig. 6h and i). A stress bending test was conducted on the quasi-solid ZAB (Fig. 6j). Despite minor voltage fluctuations under high stress, the battery promptly regains its original charging and discharging profile, highlighting its remarkable adaptability and potential as an energy source for flexible electronics.

## CONCLUSION

In summary, we establish a correlation between the ORR activity of Fe-N-C catalysts and their spin state, proposing a straightforward approach for spin regulation. Specifically, the influence of nitrogen species adjacent to Fe-N_4_ centers on the ORR activity was theoretically analyzed, showing that N_GC_ can regulate the spin state of Fe sites to enhance activity. This structure was then synthesized via a simple secondary annealing process, leveraging the different thermal stabilities of diverse nitrogen species. Systematic structural characterizations confirmed the successful fabrication of the Fe-N_4_-C catalyst with adjacent N_GC_. The Fe-N_4_/N_GC_-C catalyst possesses a higher number of unpaired electrons, which can penetrate the anti-bonding π* orbitals of O₂ to efficiently activate the reactant. The *OH intermediate at the Fe sites exhibits a longer, more easily desorbed bond, enhancing the overall reaction efficiency. Consequently, the Fe-N_4_/N_GC_-C catalyst exhibits significantly improved ORR activity. The evolution of oxygen-containing species was monitored using a range of *in-situ* techniques, which aligned well with theoretical predictions. To validate its practical application, the Fe-N_4_/N_GC_-C catalyst was employed in ZABs, demonstrating superior performance compared to batteries using a 20% Pt/C catalyst. This work offers valuable insights into the spin-dependent activity of Fe-N-C catalysts and presents an alternative strategy to optimize noble-metal-free electrocatalysts.

## Supplementary Material

nwaf061_Supplemental_Files
