# Supplementary material for: Spin-regulated Fe-N-C catalyst enabled by adjusting coordination nitrogen species for robust oxygen reduction
Source: Natl Sci Rev. 2025 Feb 20;12(7):nwaf061. doi: 10.1093/nsr/nwaf061 (PMC12153717; doi:10.1093/nsr/nwaf061)
Supplement: nwaf061_Supplemental_Files [file nwaf061_supplemental_files.zip › Supplementary data.pdf]

Spin regulated Fe-N-C catalyst enabled by adjusting coordination nitrogen species for robust oxygen reduction

Ning Wang,[a] *Chao Meng,*[a] *Bin Wang,*[a] *Xiaojie Tan,*[a] *Yi Wan,*[a] *Yang Yang,*[a] *Deyu Kong,*[a] *Wanli Wang,*[a] *Fengliang Cao,*[a] *Alistair J. Fielding,*[b] *Lina Li,*[c] *Mingbo Wu,*[a] *Han Hu*[a]*

[a] State Key Laboratory of Heavy Oil Processing, Institute of New Energy, College of Chemistry and Chemical Engineering, China University of Petroleum (East China), Qingdao, 266580, China

[b] Centre for Natural Products Discovery, School of Pharmacy and Biomolecular Sciences, Liverpool John Moores University, Byrom Street, Liverpool L3 3AF, UK

[c] Shanghai Synchrotron Radiation Facility (SSRF), Zhangjiang Lab, Shanghai Institute of Applied Physics, Shanghai Advanced Research Institute, Chinese Academy of Sciences, Shanghai 201204, China

Email: hhu@upc.edu.cn

S1. Supplemental Experimental Procedures

S1.1. Chemical and reagents

The reagents used in this work were all of analytical grade, sourced from Aladdin Co., Ltd. and utilized without further purification.

S1.2. Synthesis of electrocatalyst

Synthesis of Fe-ZIF-8 and ZIF-8

Zinc (II) nitrate hexahydrate (1.68 g) and Iron (III) nitrate nonahydrate (0.06 g) were dissolved in 150 mL of methanol for 30 minutes to achieve a transparent solution. This solution was subsequently introduced into another 150 mL methanol solution containing 2-methylimidazole (1.97 g), while maintaining continuous magnetic stirring. After 2 hours of stirring, a milky and homogeneous dispersion was formed. The dispersion was then subjected to heating at 60 ^{o}C for 12 hours, followed by cooling to ambient temperature. The precipitates were isolated by centrifugation, washed with methanol three times, and finally dried under vacuum at 60 ^{o}C for 12 hours. ZIF-8 synthesis was conducted using an identical method to that of Fe-ZIF-8, with the sole modification being the exclusion of Iron (III) nitrate nonahydrate from the reaction mixture.

Synthesis of electrospun nanofibers

Polyacrylonitrile (PAN, 0.28 g), Polyvinyl pyrrolidone (PVP, 0.14 g), and Fe-ZIF-8 (0.7 g) were mixed in N, N-dimethylformamide (DMF) with vigorous stirring at room temperature for 10 hours to prepare the dispersion for electrospinning. Subsequently, Fe-ZIF-8/PAN composite nanofibers (Fe-ZIF-8-NFs) were electrospun onto a roller collector rotating at 160 rpm using the aforementioned dispersion. The setup maintained a fixed distance of 15 cm between the spinning needle and the collector, with a flow rate of 1 mL per hour and an applied voltage of 20 kV. For comparative analysis, Fe-ZIF-8 was replaced with ZIF-8 in the dispersion for electrospinning under the identical condition.

Synthesis of CNFs catalysts ($Fe-N_4/N_{GC}-C$, $Fe-N_4-C$ and CNFs)

To produce the atomic dispersed Fe catalyst, the electrospun nanofibers containing Fe-ZIF-8 was pre-oxidized at 280 ^oC for 2h. The $Fe-N_4-C$ catalyst was obtained by further annealing at 800 ^oC for 2h. As the preparation of $Fe-N_4/N_{GC}-C$, a further annealing 1000 ^oC is required to converted the nitrogen species into the desired configurations. The CNFs was obtained by the same method as $Fe-N_4/N_{GC}-C$ except that using the precursors containing ZIF-8 instead of Fe-ZIF-8.

To provide valuable insights into the temperature dependence of the catalyst's properties, the catalysts were also prepared by annealing at 900 ^oC and 1100 ^oC, while keeping other preparation conditions unchanged.

S1.3. Characterizations

The microstructures and morphologies of the samples were observed using scanning electron microscopy (SEM) (JSM-7900F, Japan) and transmission electron microscopy (TEM) (JEM-2100F, Japan). High-angle annular dark-field scanning transmission electron microscopy (HAADF-STEM) images were recorded on a spherical aberration-corrected FEI (Titan Cubed Themis Z) operated at 300 kV with a beam current of 50 pA. The chemical composition and element mapping analysis of samples were verified using an energy dispersive X-ray spectrometer (EDS) integrated into the TEM. The thermal stability of catalyst was assessed using a Thermogravi -metric analyzer (TG, STA449F3) by heating to 1000 ^oC under nitrogen (N_2) atmosphere. The crystalline structures of the as-prepared samples were recorded on the X-ray diffractometer (XRD) (X'Pert PRO MPD, Holland) equipped with Cu Kα radiation (λ = 1.5406 Å) at an accelerating voltage of 40 kV while maintaining the emission current at 40 mA. The spectra of X-ray photoelectron spectroscopy (XPS) were recorded using an XPS spectrometer (Thermo Scientific Escalab Xi+, USA) with Al Kα radiation (1486.68 eV) as the excitation source. The Fe loading was quantified by an inductively coupled plasma optical emission spectroscopy (ICP-OES, AGILENT730). Raman spectra were acquired using a confocal laser micro-Raman spectrometer (Renishaw inVia Reflex, UK) with an excitation wavelength of 532 nm. X-ray absorption fine structure spectroscopy (XAFS) at the Fe K-edge was acquired

at the BL17B beamline platform of the Shanghai Synchrotron Radiation Facility, China. A Si (111) double-crystal monochromator was employed and the data collection was carried out in transmission mode. The acquired EXAFS data was processed following the standard procedures utilizing the ATHENA module implemented in the IFEFFIT software packages. The EXAFS spectra were obtained by subtracting the post-edge background from the overall absorption and subsequently normalizing with respect to the edge-jump step. The quantitative structural parameters around central atoms were analyzed using the ARTEMIS module of IFEFFIT software packages. Electron paramagnetic resonance (EPR) spectra were collected on a Bruker EMXplus spectrometer operating at ambient temperature. The temperature-dependent magnetic susceptibility measurements were performed on a physical property measurement system (PPMS, Quantum Design) with a magnetic field strength of 1000 Oe over the temperature range from 2 to 300 K. Attenuated total reflectance surface-enhanced infrared absorption spectroscopy (ATR-SEIRAS) measurements were conducted using a Thermo Scientific Thermo 8700 equipped with a liquid-nitrogen cooled MCT−A detector.

S1.4. Electrochemical measurements

Electrochemical performance evaluations were conducted in 0.1 M KOH solution using a CHI 760E electrochemical workstation (CH Instruments, Inc.) with a rotation-control equipment (Pine Research Instrument, USA) in a standard three-electrode system. Rotating ring-disk electrode (RRDE) with a glassy carbon disk (GC, diameter of 5.61 mm, Pine Research Instrumentation) was applied as working electrode, while the saturated calomel electrode (SCE) and Pt wire served as the reference electrode and counter electrode, respectively. The potentials versus SCE were normalized to the reversible hydrogen electrode (RHE) using the Nernst Equation (1):

$$E_{\mathrm{RHE}} = E_{\mathrm{SCE}} + 0.0591 \times \mathrm{pH} + 0.241 \quad (1)$$

To prepare catalyst ink, catalyst (2 mg) and 5 wt% Nafion solution (5 μL) were mixed in ethanol (800 μL) for 1 h. Then, 30 μL of the catalyst ink was spread on the

GC with a loading amount of 0.30 mg cm^{-2}. Consequently, Pt/C based ink was prepared using the same procedure and employed as the reference catalyst for ORR evaluation.

The ORR performance was recorded at ambient temperature and the electrolyte was purged with high-purity N_2 or O_2 for a minimum duration of 30 minutes before the measurements. Continuous gas flow was maintained throughout the experimental procedure. The cyclic voltammetry (CV) and linear sweep voltammetry (LSV) curves were recorded at scan rates of 50 mV s^{-1} and 5 mV s^{-1}, respectively.

Tafel slopes were determined using the Tafel Equation (2) based on the obtained LSV data, where *j* is the current density:

$$\eta = blog(j) + a \tag{2}$$

The corresponding electron transfer number (*n*) was calculated by applying the Koutecky–Levich (K-L) Equation (3) and (4) at various rotation speeds from 400 to 2025 rpm:

$$\frac{1}{j} = \frac{1}{j_L} + \frac{1}{j_K} = \frac{1}{B\omega^{1/2}} + \frac{1}{j_K} \tag{3}$$

$$B = 0.62nFC_0(D_0)^{2/3}\nu^{-\frac{1}{6}} \tag{4}$$

where *j* represents the measured current density (mA cm^{-2}), the term of j_L and j_K correspond to the diffusion-limiting current density (mA cm^{-2}) and the kinetic current density (mA cm^{-2}), respectively, ω denotes the angular velocity of the disk (rad s^{-1}), *n* is the electron transfer number. While the value of constant B is related to a series of parameter including Faraday constant (F, 96485 C mol^{-1}), the diffusion coefficient of O_2 in 0.1 M KOH (D_0, 1.9×10^{-5} cm^2 s^{-1}), the bulk concentration of O_2 (C_0, 1.2×10^{-3} mol L^{-1}), and the kinetic viscosity of the electrolyte in 0.1 M KOH (ν, 1.13×10^{-2} cm^2 s^{-1}).

For RRDE, the collection efficiency of the Pt ring (N) is 0.37, the percentage of hydrogen peroxide formation yields (H_2O_2 %) and the electron transfer number (n) were evaluated by the following Equations (5) and (6):

$$n = \frac{4 \times I_d}{I_d + I_r/N} \tag{5}$$

$$H_2O_2(\%) = 200 \times \frac{I_r/N}{I_d + I_r/N} \quad (6)$$

where I_d is the disk current, I_r is the ring current.

The CV curves obtained at different scan rates (2, 4, 6, 8, and 10 mV s^{-1}) in a non-Faradaic region were utilized for the determination of double layer capacitance (C_{dl}) of the catalyst. The electrochemical active surface area (ECSA) is calculated through the Equation (7):

$$\text{ECSA} = \frac{C_{dl}}{C_s} \quad (7)$$

The mass activity (MA) of the catalyst was determined using the Equation (8):

$$\text{MA (mA mg}^{-1}) = \frac{j}{C_{cat}} \quad (8)$$

where j is the measured current density under a potential (V vs. RHE) and *C*cat denotes the quality per unit area of the catalyst.

The Specific Activity (SA) is calculated through the Equation (9):

$$\text{SA (mA cm}^{-1}) = \frac{j}{ECSA} \quad (9)$$

where j is the measured current density under a potential (V vs. RHE).

The turn-over frequency (TOF) is estimated by the Equation (10):

$$\text{TOF [e/(site} \cdot \text{s)]} = \frac{j_K}{n_{metal} \times F} = \frac{j_K \times M_{metal}}{m_{metal} \times F} = \frac{j_K \times M_{metal}}{m_0 \times \omega_{metal} \times F} \quad (10)$$

Where j_K is the kinetic current density, m_0 is the mass of catalyst loading on the electrode, ω_{metal} is the metal content in the catalyst, F is the Faraday constant (96485 C mol^{-1}), and n_{metal}, m_{metal}, and M_{metal} represent the mol amount, mass, and atomic weight of metal in the catalysts, respectively.

The *in-situ* Electrochemical impedance spectroscopy (EIS) spectra, *in-situ* ATR-SEIRAS, and in-situ Raman were recorded by coupling the electrochemical testing with a CHI 760E electrochemical workstation under various potentials in an O_2-saturated 0.1 mol KOH solution. The EIS spectra were acquired over a frequency range of 0.1 Hz to 0.1 MHz and subsequently fitted to the equivalent circuit model employing commercially available software (ZView, Scribner Associates Inc).

S1.5. Zn-air batteries (ZABs) measurement

Assembly of ZABs with liquid electrolytes:

A ZAB was constructed using a polished Zn plate with a thickness of 0.5 mm as the anode, a catalyst-coated carbon cloth as the air cathode, and an aqueous solution containing KOH (6 M) with $Zn(CH_3COO)_2$ (0.2 M) as the electrolyte. The catalyst ink was coated onto the carbon cloth with a loading amount of 1mg cm^{-2}.

Assembly of the quasi-solid ZABs:

A polished zinc foil served as the anode, and $Fe\text{-}N_4/N_{GC}\text{-}C$ was utilized as the air cathode catalyst. Sodium polyacrylate (PANa) hydrogel was employed as the electrolyte. The preparation of the PANa electrolyte gel is detailed below: 7 mL of 14.3 M NaOH solution and 6.8 mL of aqueous acrylic monomer solution were added to 6.8 mL of deionized water and stirred at 5 °C for 10 minutes. Then, 110 mg of ammonium persulfate were introduced as an initiator. After that, 4.2 mL of N, N'-dimethylacrylamide was added dropwise and the mixture was stirred at room temperature for 30 minutes. Finally, the homogenized solution was poured into a petri dish and dried at 70 °C for 1 hour.

Electrochemical evaluation of the ZABs：

The charge and discharge polarization curves were measured with CHI 1140E electrochemical workstation at a scan rate of 10 mV s^{-1}. The specific discharge capacity was normalized by the consumed mass of Zn plate during the long-term discharge process. The durability was evaluated by charging and discharging at a current density of 10 mA cm^{-2} for 20 mins per cycle (10 mins charge, 10 mins discharge) on the Land apparatus (CT2001A).

S1.6. Calculations of the effective magnetic moments (μ_{eff}) and the number of unpaired electrons (*n*):

The total effective magnetic moments (μ_{eff}) for samples could be calculated by using the Equation (11), in accordance with the Langevin theory,

$$\mu_{\text{eff}} = \sqrt{8C}\mu_{\text{B}} \tag{11}$$

Where C is Curie constant, derived from the fittings on the susceptibility (χ=M/H) above the paramagnetic transition temperatures, following a Curie-Weiss law $\chi=C/(T-\Theta)$. Here, Θ represents the Curie-Weiss temperature. The μ_{eff} of Fe sites originates from Fe^{3+} magnetic ion, and the volume fractions of Fe^{3+} in the high-spin (HS), medium-spin (MS), and low-spin (LS) states can be calculated from the relationship:

$$\mu_{\text{eff}} = \mathrm{g}\mu_{\text{B}}\sqrt{S_{\text{HS}}(S_{\text{HS}}+1)V_{\text{HS}} + S_{\text{MS}}(S_{\text{MS}}+1)V_{\text{MS}} + S_{\text{LS}}(S_{\text{LS}}+1)V_{\text{LS}}} \quad (12)$$

$$V_{\text{H}S} + V_{\text{M}S} + V_{\text{L}S} = 1 \quad (13)$$

In the aforementioned equations, g is the Lande factor (g = 2), while S_{HS} (= 5/2), S_{MS} (= 3/2), and S_{LS} (= 1/2) are the S values. V_{HS}, V_{MS}, and V_{LS} are the volume fractions for Fe^{3+} in HS, MS, and LS states, respectively. The number of unpaired d electron (n) of Fe^{3+} could be derived using the following Equation (14):

$$\mu_{\text{eff}} = \sqrt{n(n+2)}\mu_{\text{B}} \quad (14)$$

S1.7. DFT calculations

DFT calculations were conducted utilizing the Vienna ab initio Simulation Package (VASP) code.[1] The ion-electron interactions were characterized using projector-augmented wave (PAW) pseudopotentials,[2] while the exchange and correlation functionals were described by the Perdew-Burke-Ernzerhof (PBE) functional within the generalized gradient approximation (GGA) framework.[3] To accurately model the localized metal d-electrons in the atomically dispersed Fe catalysts, the Hubbard-U correction (DFT+U method) was implemented, with a U−J value set to 3.29 eV, based on previous literature.[4] The plane-wave cutoff energy was established at 500 eV, the convergence thresholds for energy and force on each atom were set to 10^{-5} eV and 0.01 eV Å^{-1}, respectively. The geometry optimization was performed using Monkhorst–Pack k-point meshes of 3 × 3 × 1. In addition, the Grimme method (DFT-D3) was applied to accurately account for the physical van der Waals (vdW) interactions.[5, 6] The graphene monolayer was doped with a Fe-N coordinated structure, where the thickness of the vacuum layer in the Z direction was

set to 15 Å. The electronic spin polarization was considered in all the calculations. In alkaline media, the ORR typically follows a four-electron transferred pathway:

$$O_2 + H_2O + e^- + * \rightarrow * OOH + OH^- \quad (15)$$

$$* OOH + e^- \rightarrow * O + OH^- \quad (16)$$

$$* O + H_2O + e^- + * \rightarrow * OH + OH^- \quad (17)$$

$$* OH + e^- \rightarrow OH^- + * \quad (18)$$

The free energy change of each step was determined by Equation (19):

$$\Delta G = \Delta E + \Delta E_{ZPE} - T\Delta S + \Delta G_U \quad (19)$$

In this equation, ΔE represents the total energy difference between reactants and products, directly derived from DFT calculations. ΔE_{ZPE} is the difference of zero-point energy and ΔS is the variation of entropy. ΔG_U accounts for the free energy contributions associated with the electrode potential U. The impact of electrode potential U on the elementary step involving electron and proton transfer is determined by $\Delta G_U = -neU$, where n represents the number of transferred electrons. T is the temperature (T = 298.15 K).

The free energy of H^+ is equivalent to half the free energy of hydrogen molecules (H_2):

$$G(H^+) = \frac{1}{2} G(H_2) \quad (20)$$

S2. Supplemental Figures

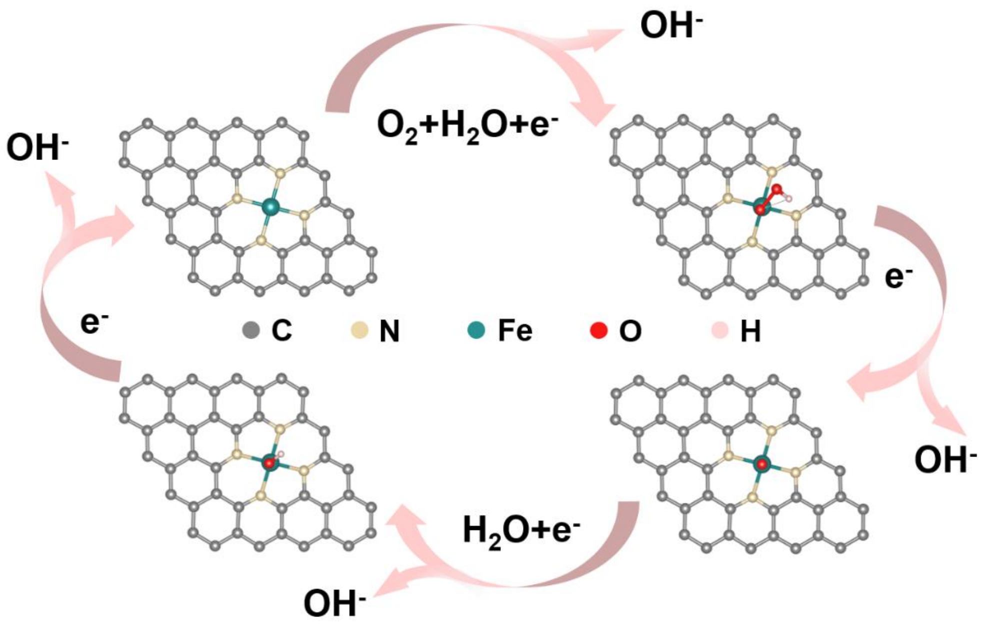

Figure S1. Reaction intermediates for ORR on the Fe-N_4-C.

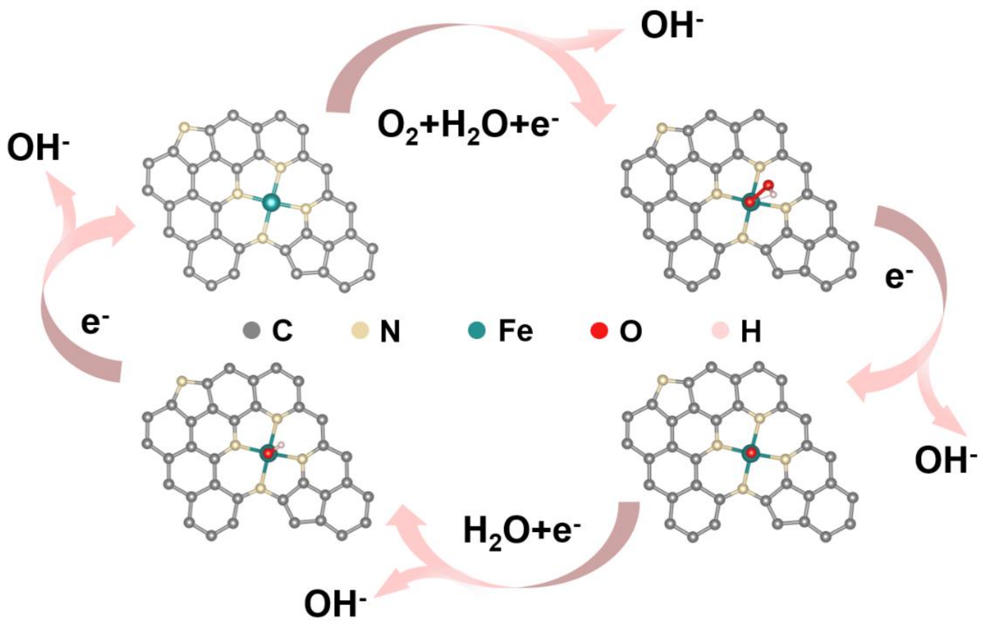

Figure S2. Reaction intermediates for ORR on the Fe-N_4/N_{PR}-C.

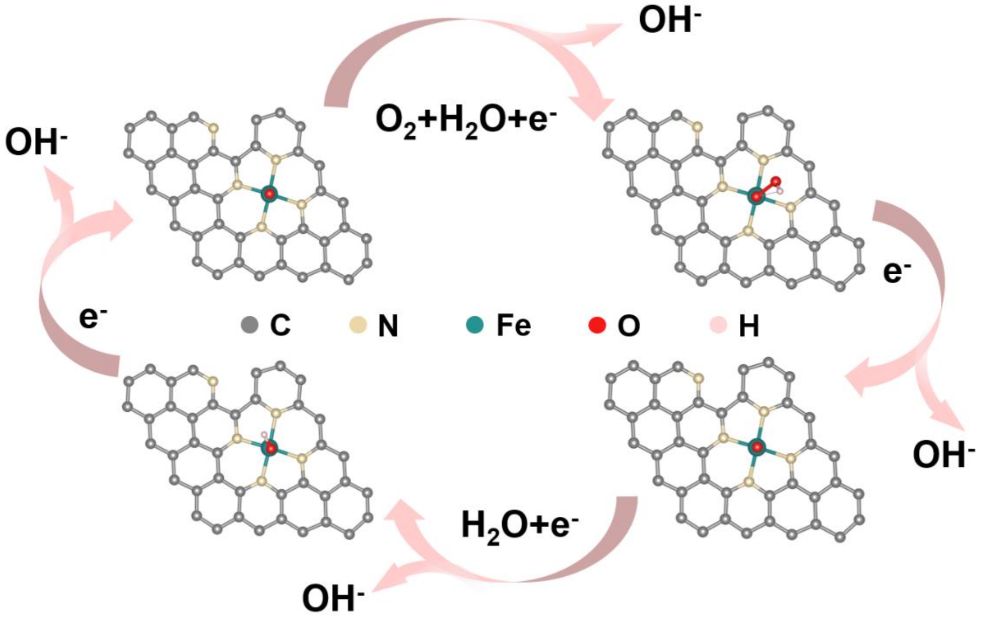

Figure S3. Reaction intermediates for ORR on the Fe-N_4/N_{PD}-C.

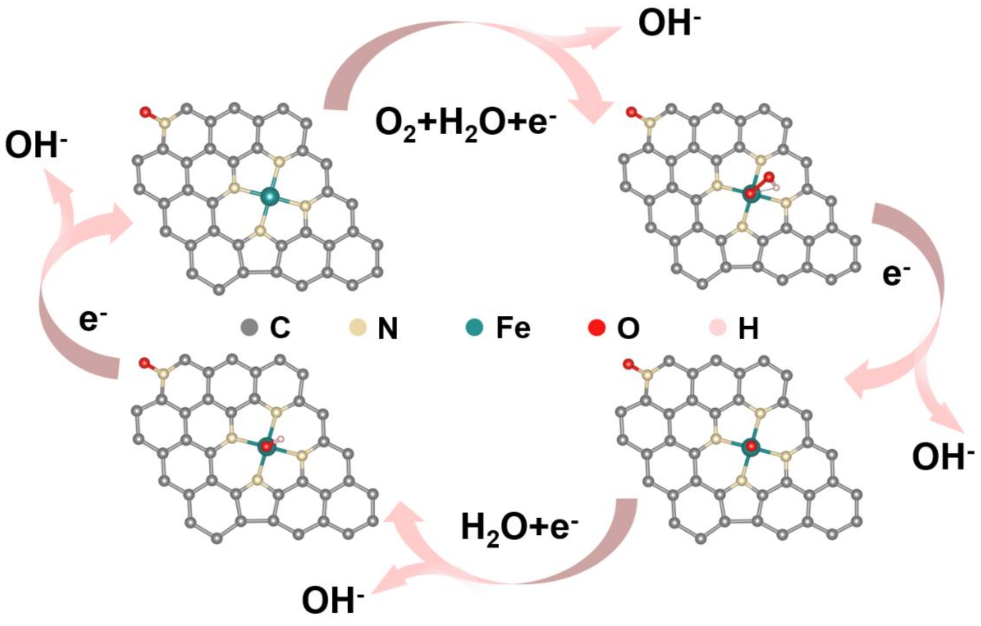

Figure S4. Reaction intermediates for ORR on the Fe-N_4/N_{OD}-C.

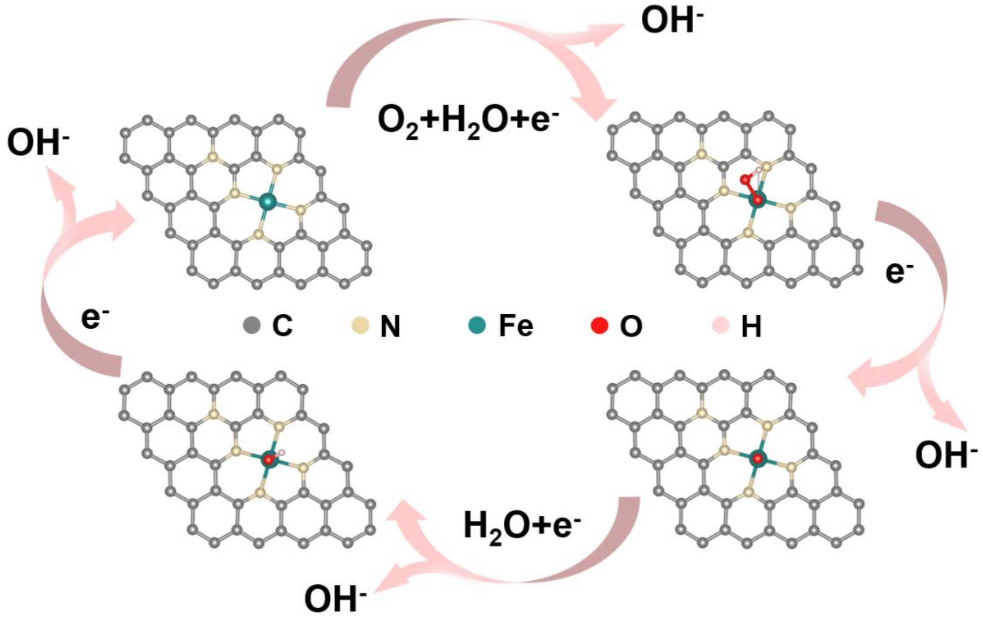

Figure S5. Reaction intermediates for ORR on the Fe-N_4/N_{GC}-C.

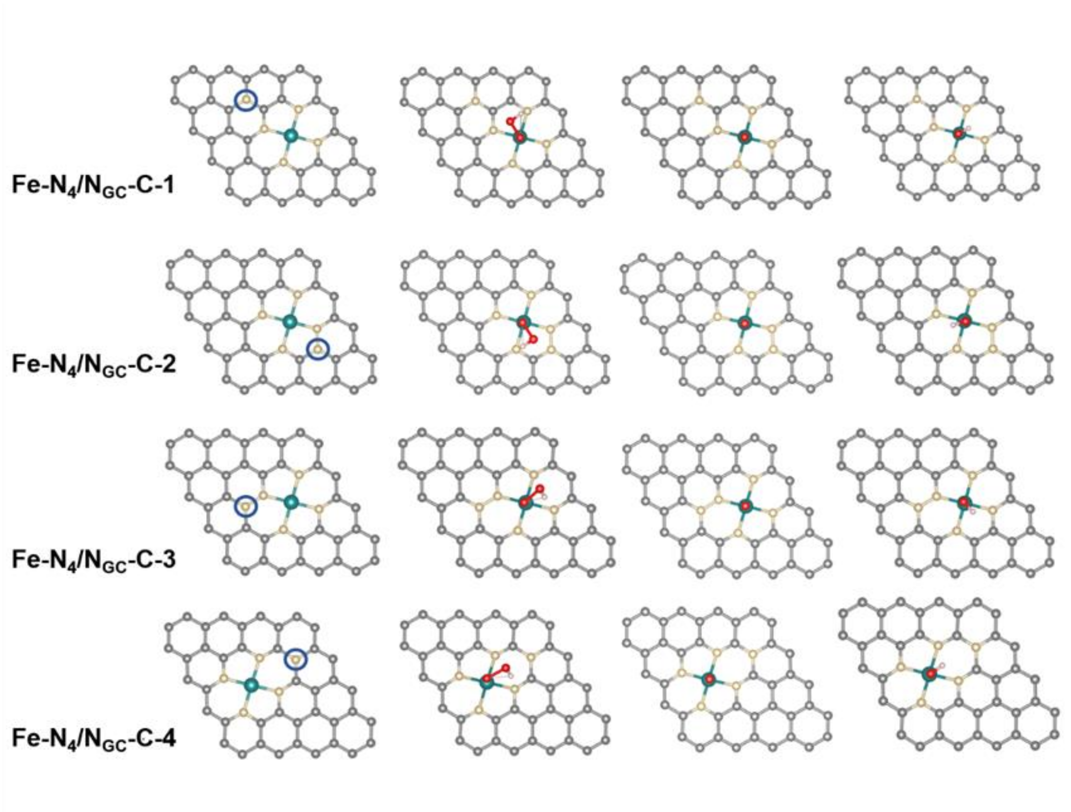

Figure S6. Illustrated structures of Fe-N_4/N_{GC}-C with graphitic nitrogen (N_{GC}) doping at different sites. (green ball: Fe; yellow ball: N; gray ball: C; red ball: O; pink ball: H).

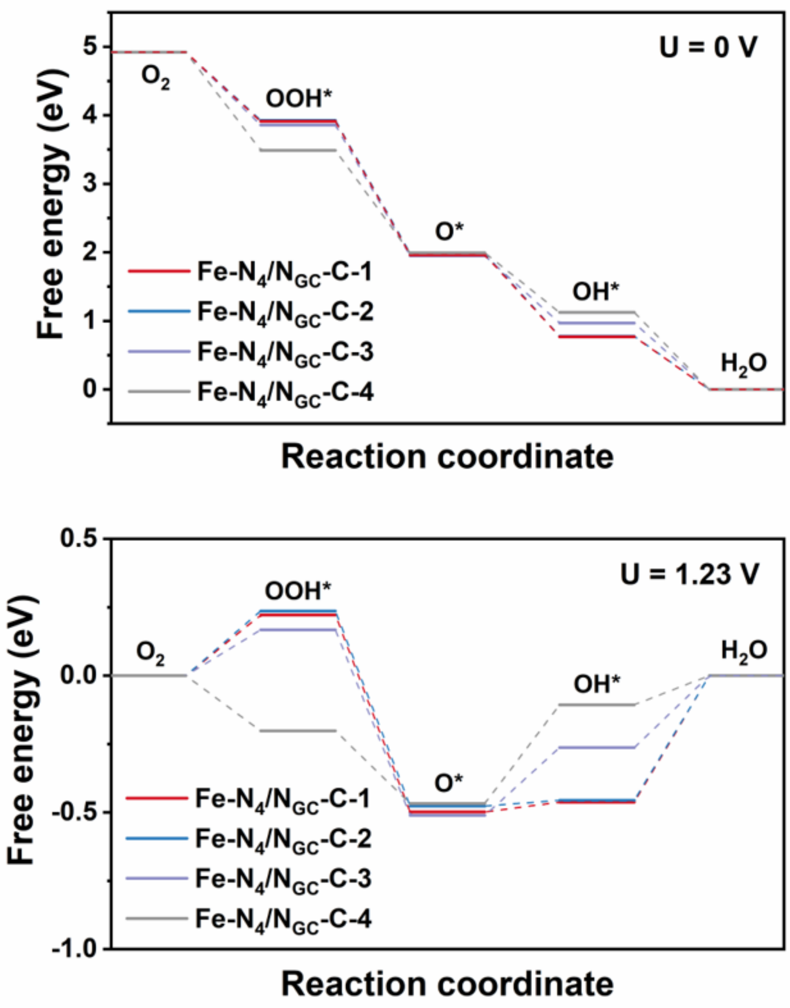

Figure S7. Gibbs free energy diagrams of ORR for Fe-N_4/N_{GC}-C with graphitic-N doped at different sites at 0 V and 1.23 V.

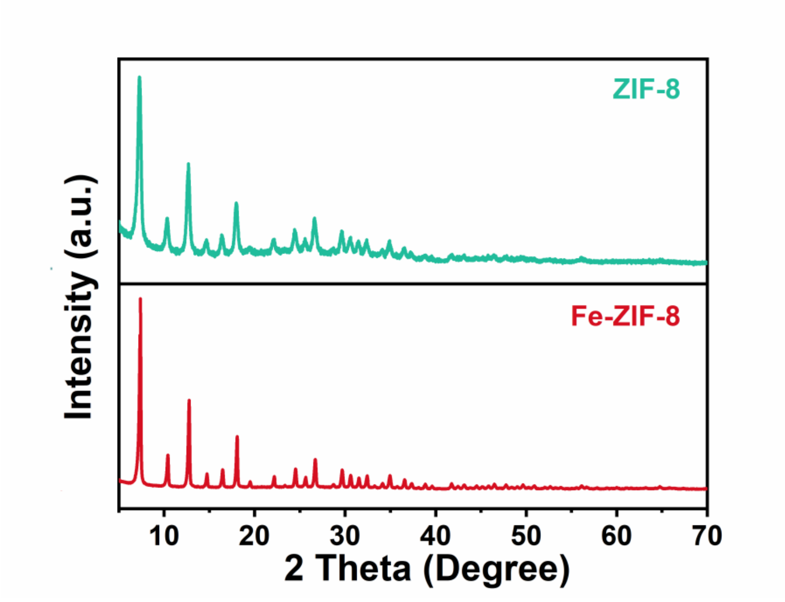

Figure S8. XRD patterns of ZIF-8 and Fe-ZIF-8.

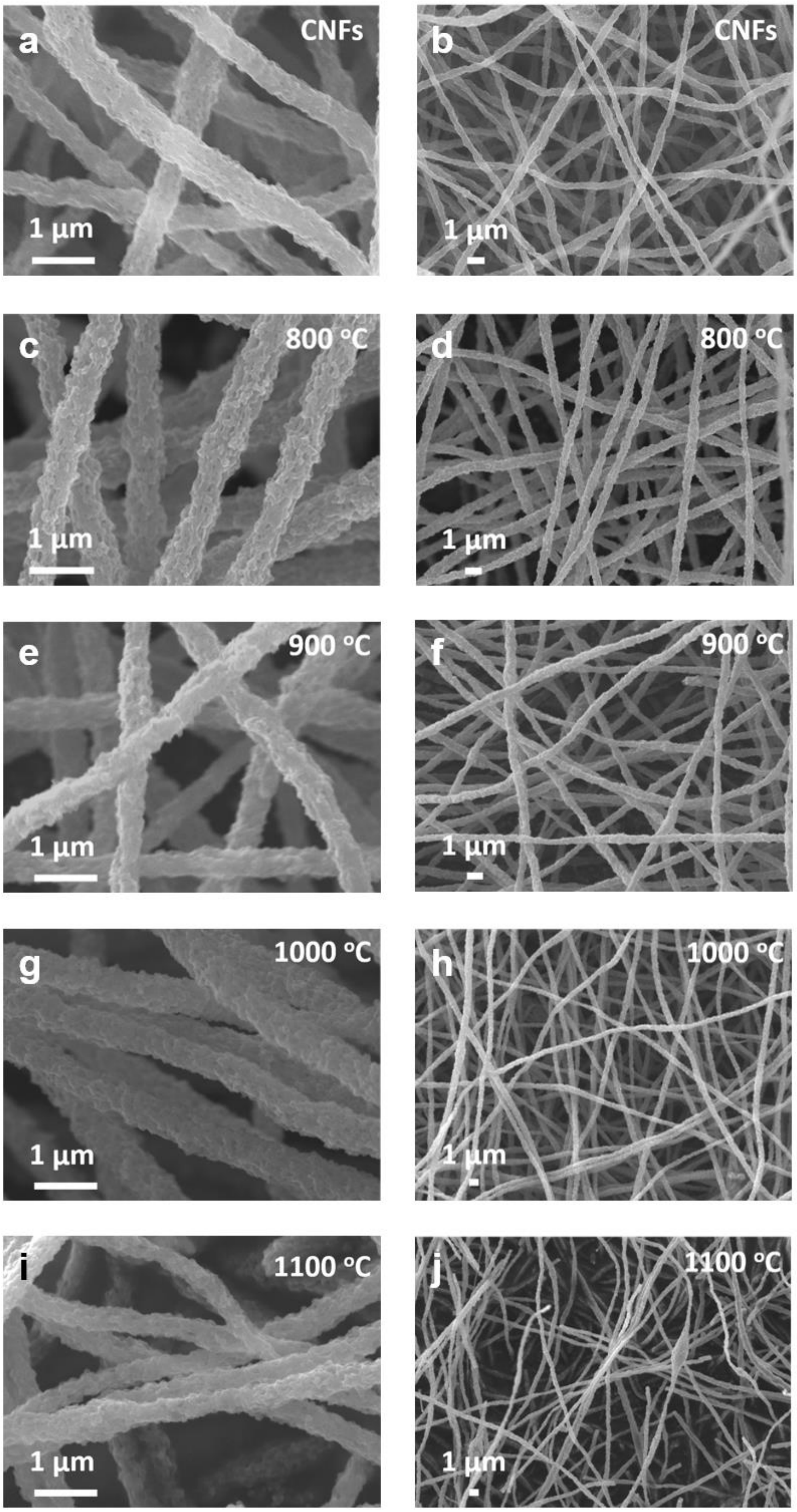

Figure S9. SEM images of (a, b) CNFs and the Fe-based nanofibers obtained at (c, d) 800 ^{o}C (Fe-N_4-C), (e, f) 900 ^{o}C, (g, h) 1000 ^{o}C (Fe-N_4/N_{GC}-C), and (i, j) 1100 ^{o}C.

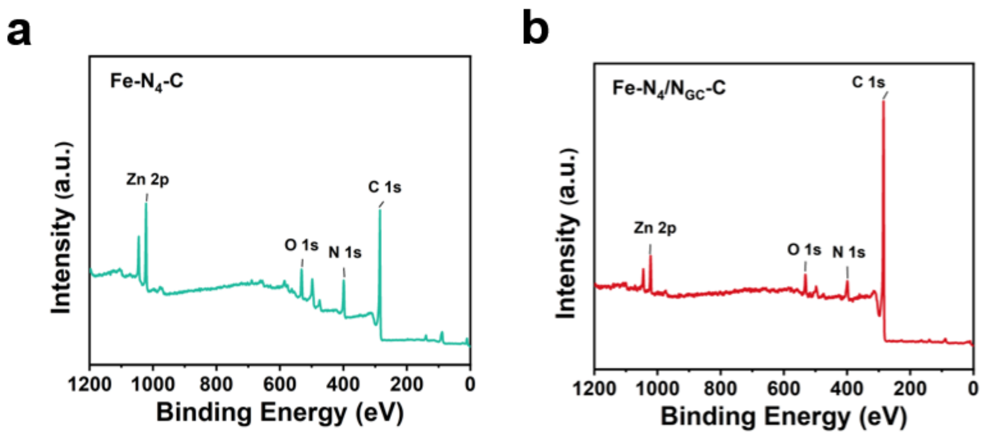

Figure S10. XPS spectra of (a) Fe-N_4-C and (b) Fe-N_4/N_{GC}-C.

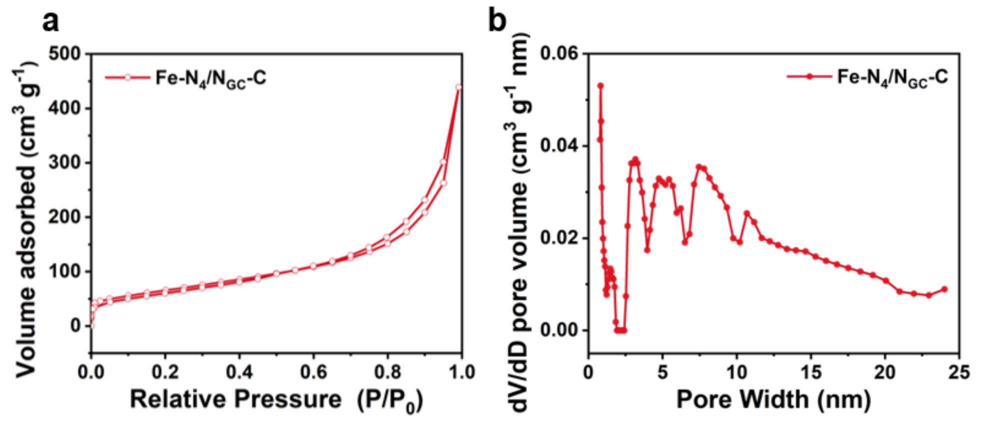

Figure S11. (a) N_2 adsorption/desorption isotherm and (b) pore size distribution of Fe-N_4/N_{GC}-C.

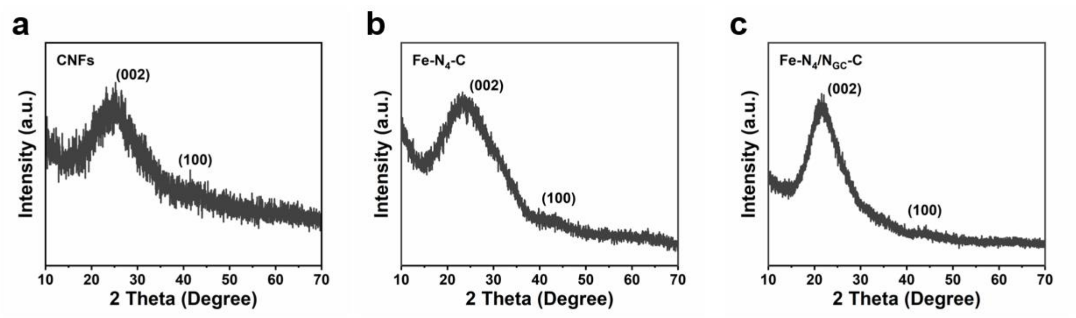

Figure S12. XRD patterns of (a) CNFs, (b) Fe-N_4-C, and (c) Fe-N_4/N_{GC}-C.

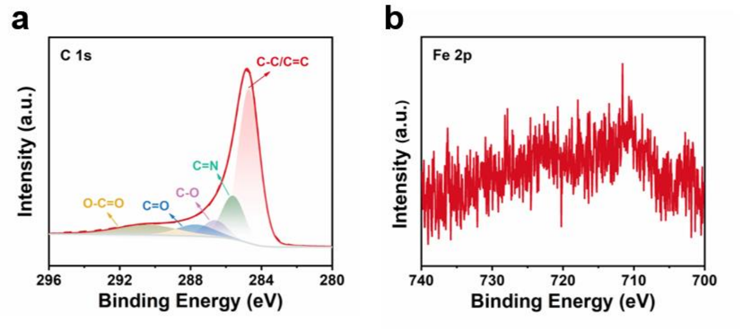

Figure S13. High-resolution XPS spectra of (a) C 1*s* and (b) Fe 2*p* of Fe-N_4/N_{GC}-C.

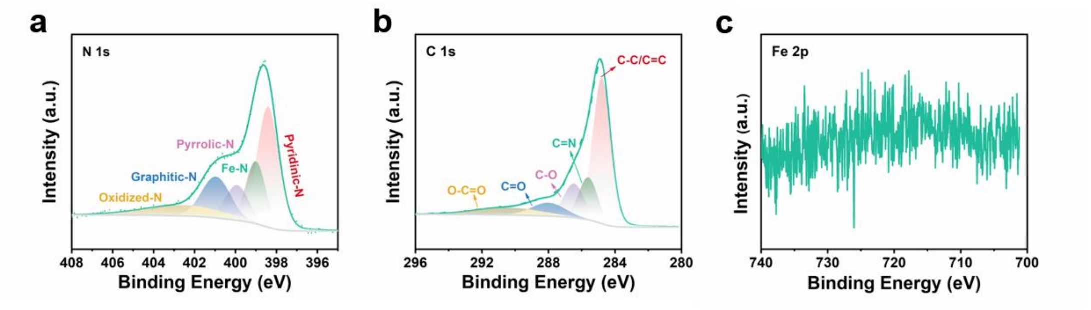

Figure S14. High-resolution XPS spectra of (a) N 1*s*, (b) C 1*s*, and (c) Fe 2*p* of Fe-N_4-C.

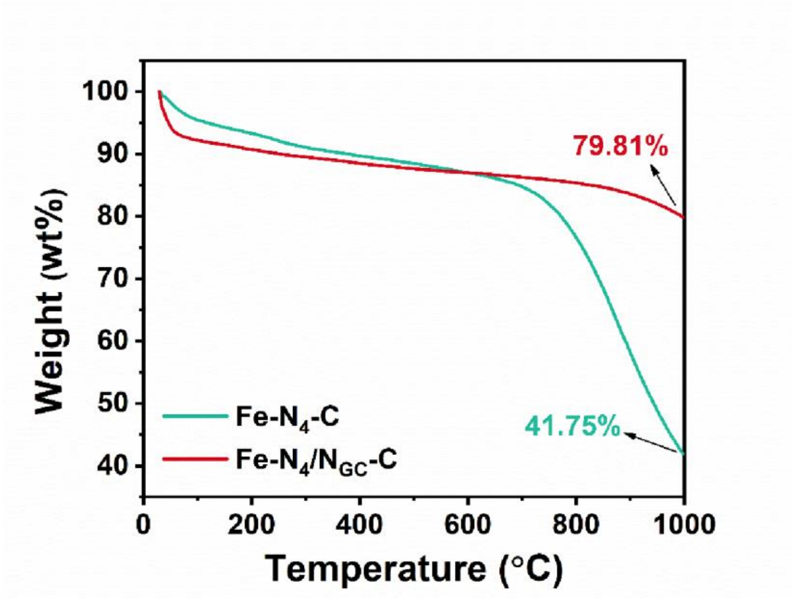

Figure S15. (a) TG curves of $Fe\text{-}N_4\text{-}C$ and $Fe\text{-}N_4/N_{GC}\text{-}C$ in an inert condition.

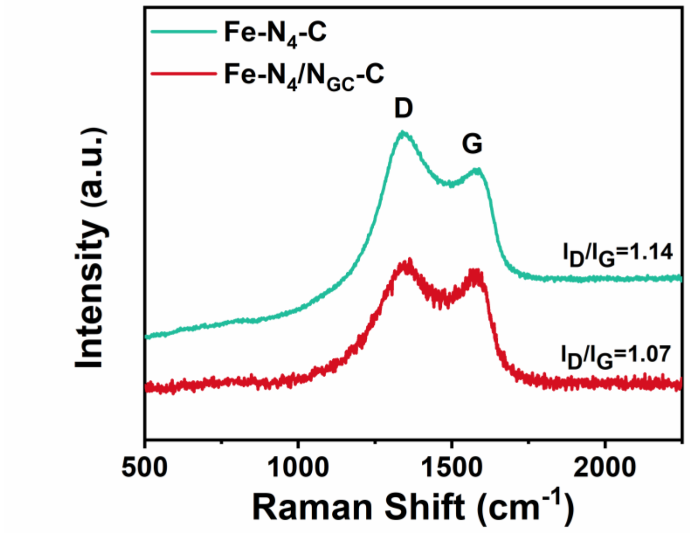

Figure S16. (a) Raman spectra of $Fe\text{-}N_4\text{-}C$ and $Fe\text{-}N_4/N_{GC}\text{-}C$.

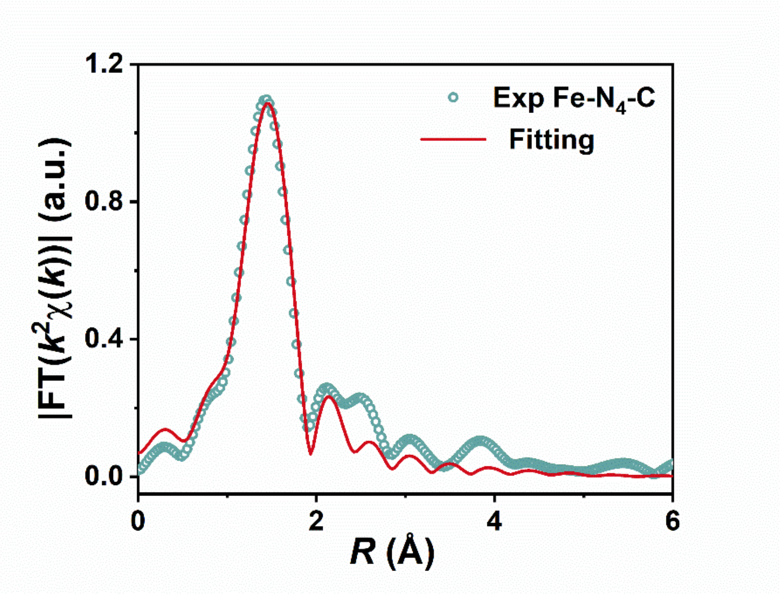

Figure S17. Corresponding FT-EXAFS fitting curve at the Fe K-edge of Fe-N_4-C.

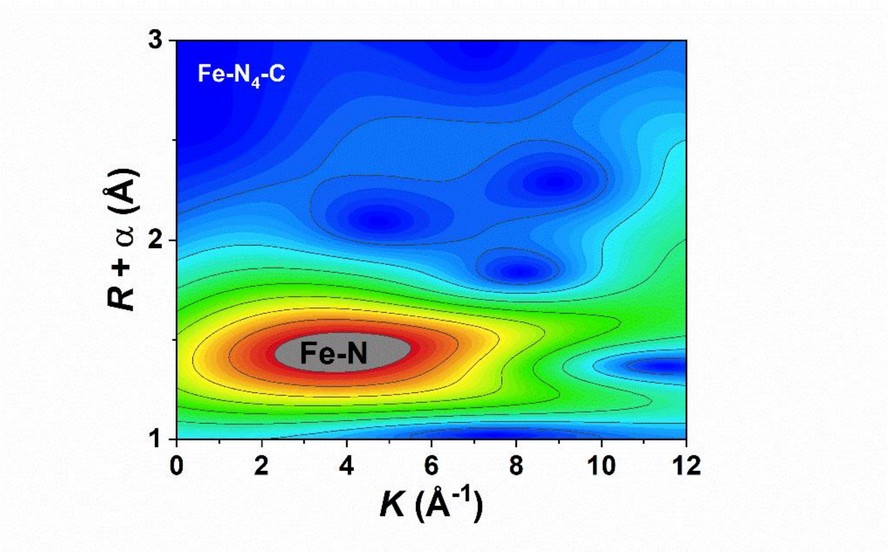

Figure S18. Wavelet transforms for the k2-weighted Fe K-edge EXAFS signals for Fe-N_4-C.

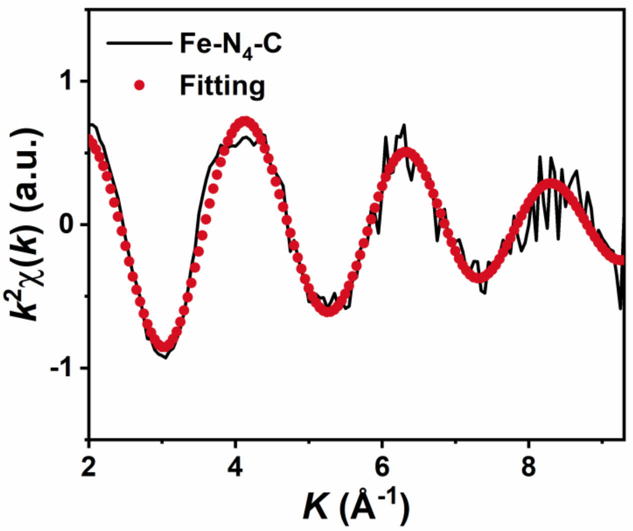

Figure S19. K space curve of Fe-N_4-C and corresponding fitting curve.

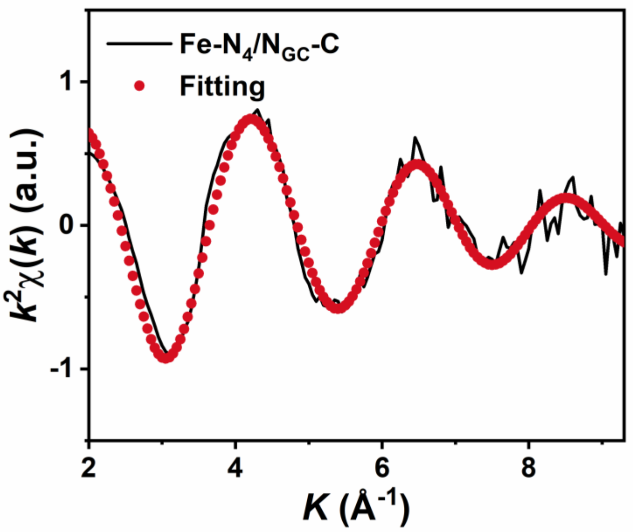

Figure S20. K space curve of Fe-N_4/N_{GC}-C and corresponding fitting curve.

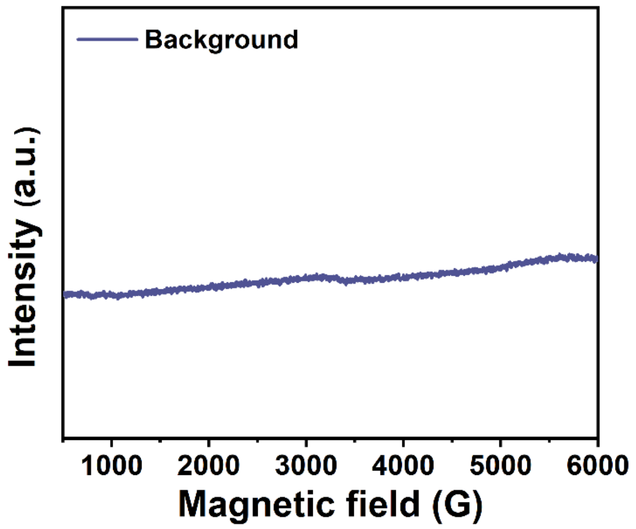

Figure S21. Background from the sample holder (empty tube).

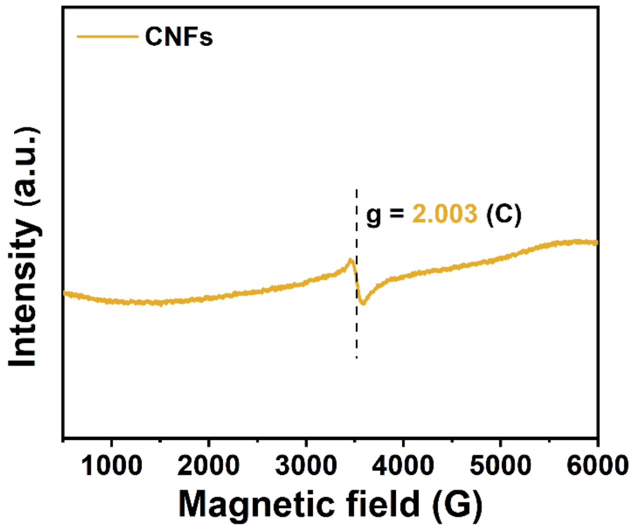

Figure S22. The mass-normalized EPR signal of CNFs.

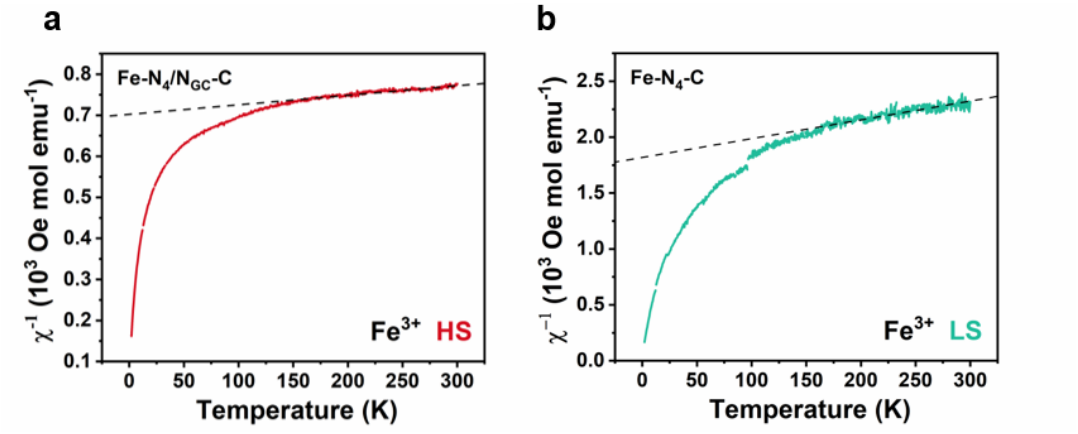

Figure S23. 1/χ plots for the (a) Fe-N_4/N_{GC}-C and (b) Fe-N_4-C.

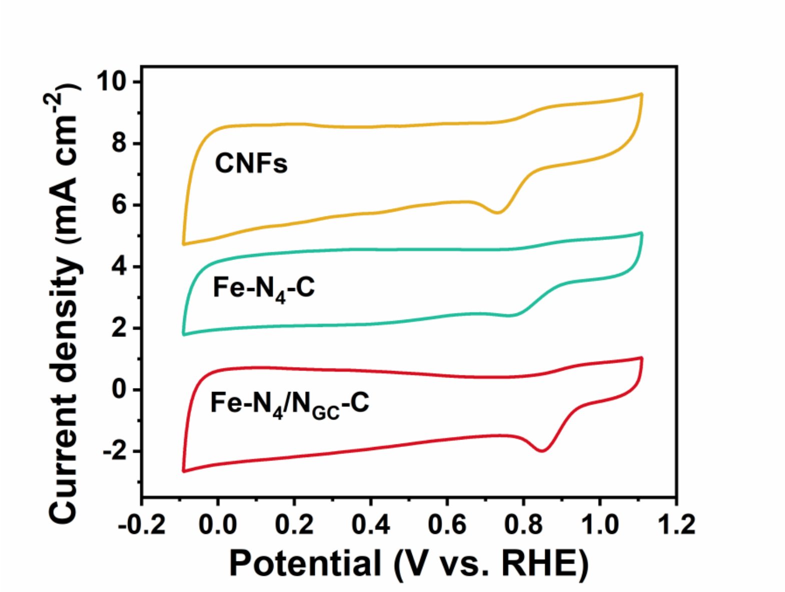

Figure S24. CV curves of the catalysts in O_2-saturated 0.1 M KOH solution.

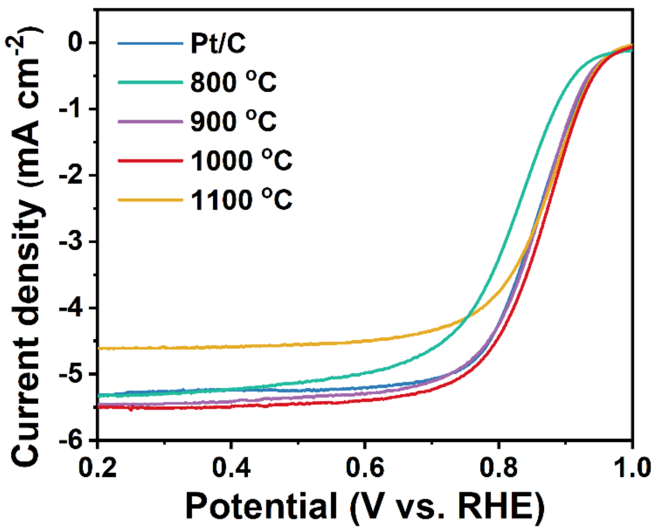

Figure S25. LSV curves of the catalysts prepared at different temperatures in O_2-saturated 0.1 M KOH solution.

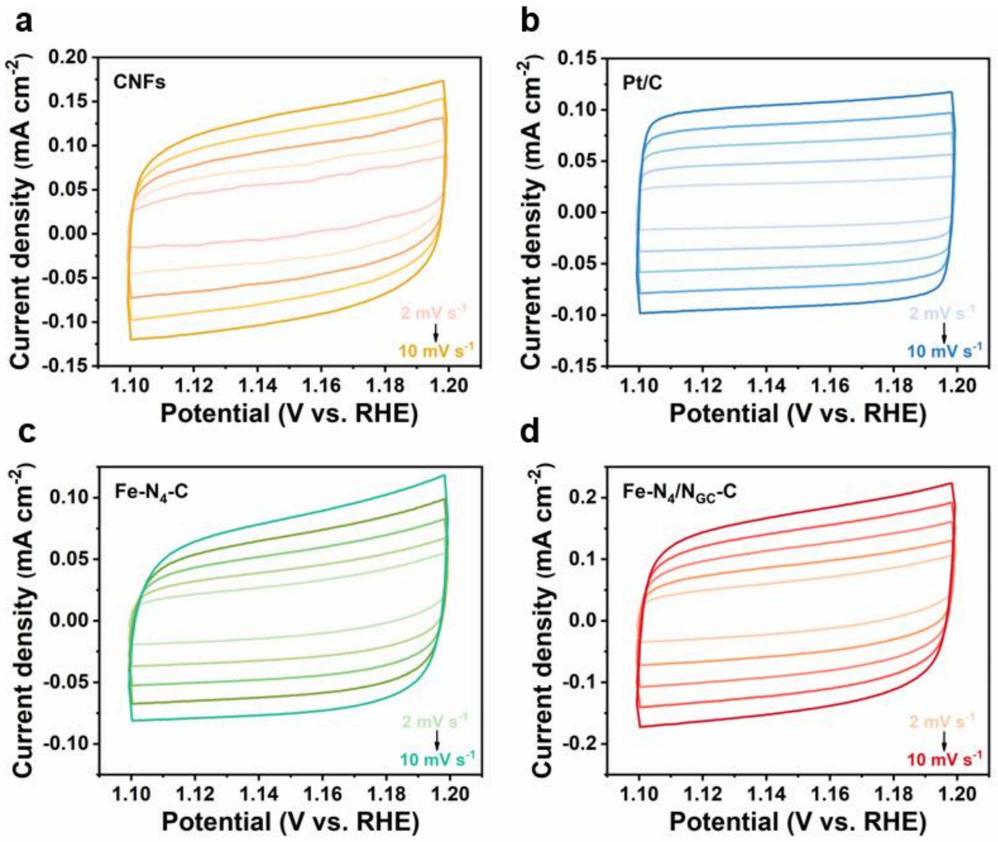

Figure S26. CV curves at scan rates from 2 to 10 mV s^{-1} for (a) CNFs, (b) Pt/C, (c) $Fe-N_4-C$, and (d) $Fe-N_4/N_{GC}-C$ collected in 0.1 M O_2-saturated KOH solution.

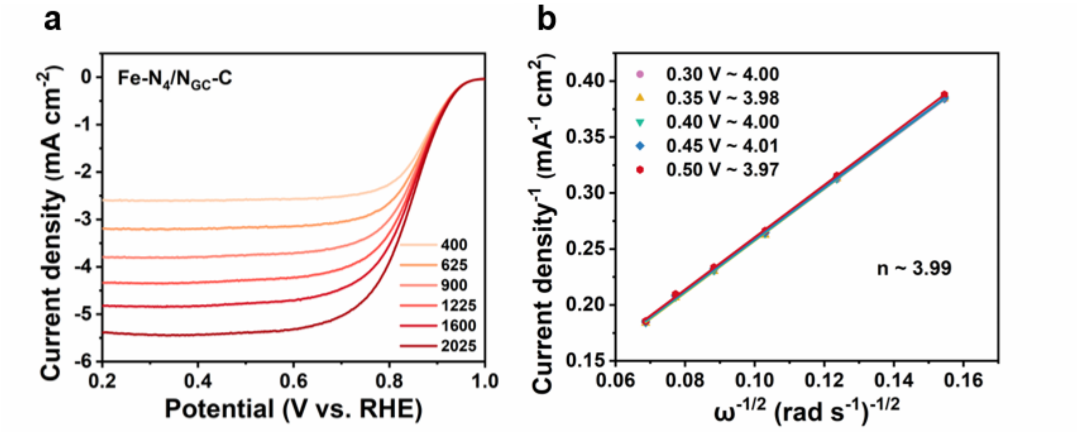

Figure S27. (a) LSV curves of Fe-N_4/N_{GC}-C at different rotating speeds from 400 to 2025 rpm in 0.1 M O_2-saturated KOH solution. (b) The corresponding K–L plots at various potentials.

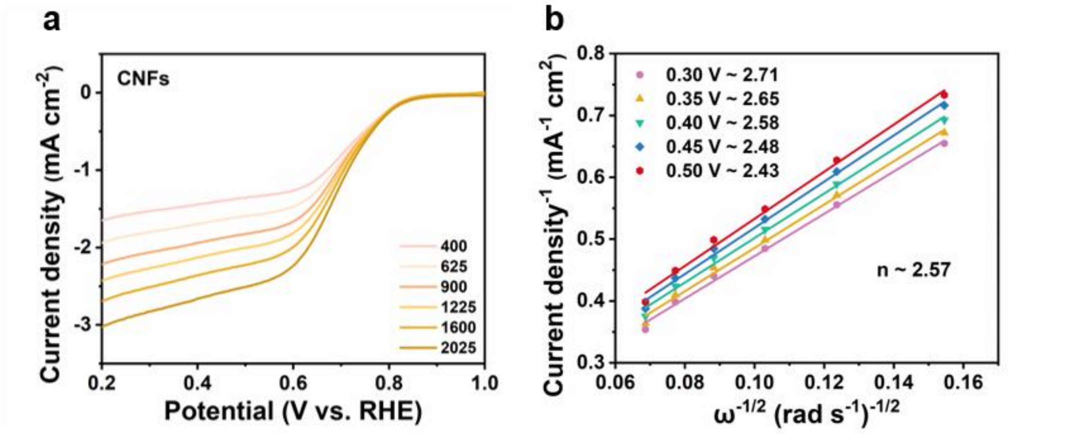

Figure S28. (a) LSV curves of CNFs at different rotating speeds from 400 to 2025 rpm in 0.1 M O_2-saturated KOH solution. (b) The corresponding K–L plots at various potentials.

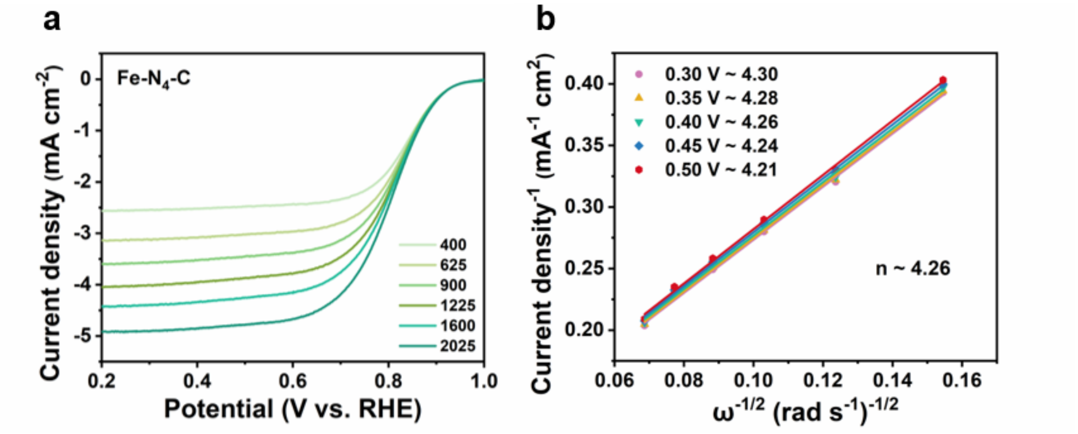

Figure S29. (a) LSV curves of Fe-N_4-C at different rotating speeds from 400 to 2025 rpm in 0.1 M O_2-saturated KOH solution. (b) The corresponding K–L plots at various potentials.

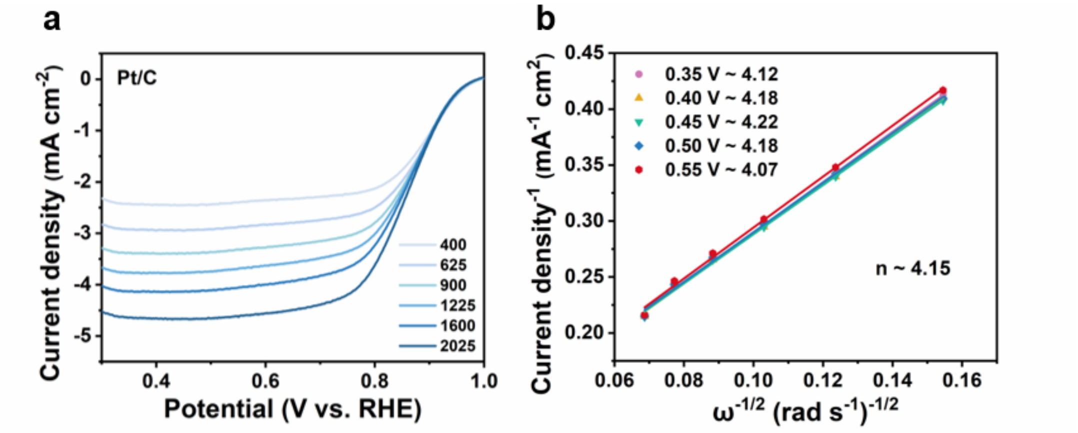

Figure S30. (a) LSV curves of Pt/C at different rotating speeds from 400 to 2025 rpm in 0.1 M O_2-saturated KOH solution. (b) The corresponding K–L plots at various potentials.

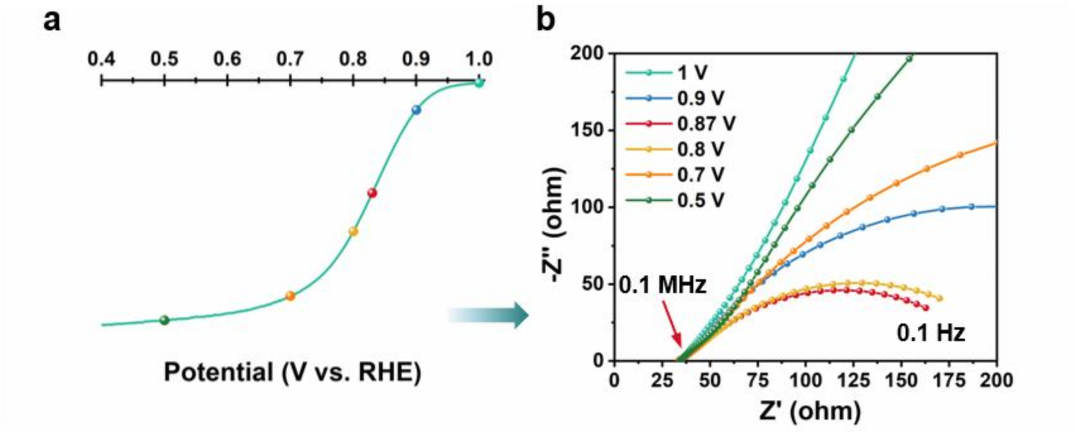

Figure S31. (a) The LSV curve of the Fe-N_4-C at 1600 rpm. (b) Nyquist plots of ORR on Fe-N_4-C.

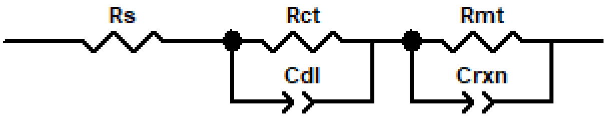

Figure S32. The equivalent circuit was used for modeling the measured electrochemical response.

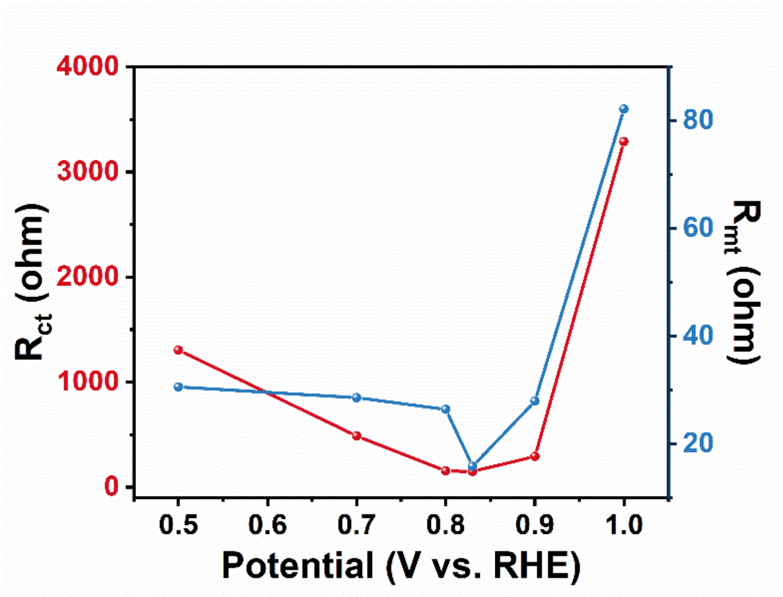

Figure S33. Response of the R_{ct} and R_{mt} at different potentials for Fe-N_4-C.

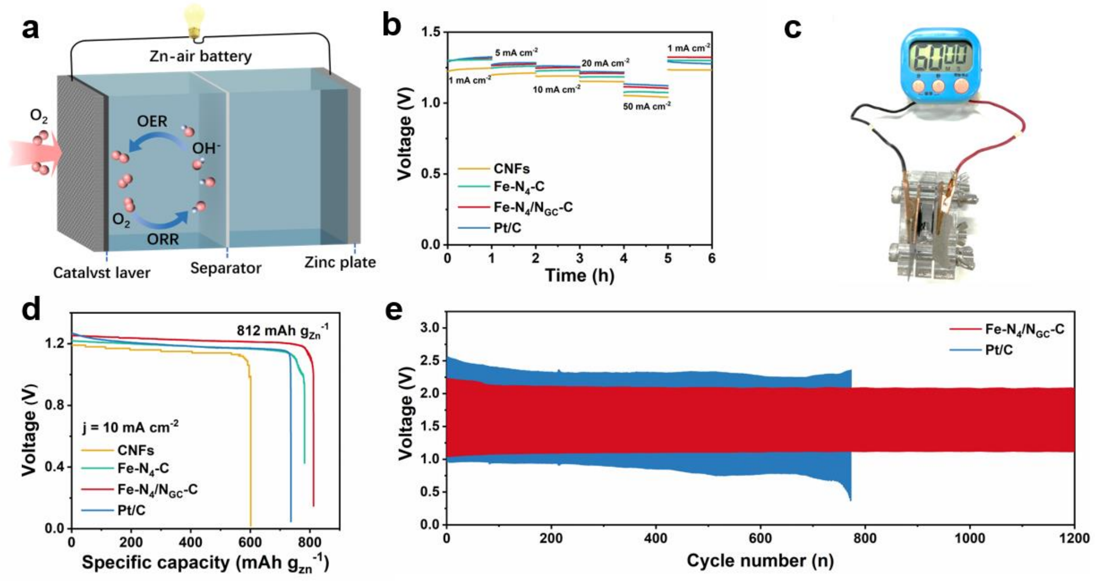

Figure S34. The electrochemical performance of ZABs using a liquid electrolyte: (a) structural illustration of the ZAB. (b) Discharge plots at different current densities. (c) The electronic timer powered by a ZAB using Fe-N_4/N_{GC}-C as the air cathode catalyst. (d) Specific capacity plots of ZABs using different catalysts at 10 mA cm^{-2}. (e) Galvanostatic long-term charge and discharge profiles at 10 mA cm^{-2}.

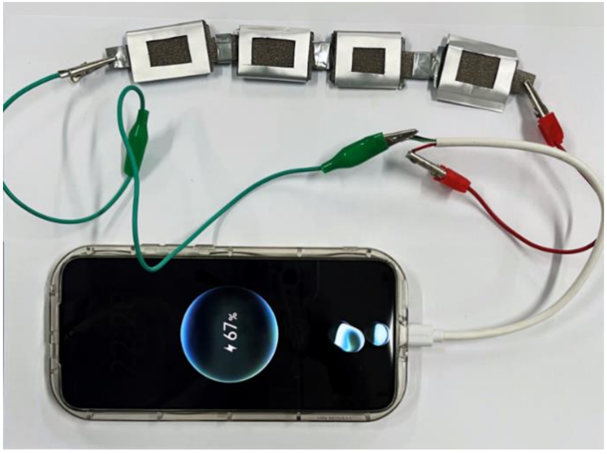

Figure S35. Photograph of a mobile phone powered by four series-connected quasi-solid ZABs.

Table S1. Calculated Gibbs free energies of ORR intermediates for $Fe-N_4-C$, $Fe-N_4/N_{BR}-C$, $Fe-N_4/N_{BD}-C$, $Fe-N_4/N_{ON}-C$, and $Fe-N_4/N_{GC}-C$ at U=0 V.

	$Fe-N_4-C$	$Fe-N_4/N_{PR}-C$	$Fe-N_4/N_{PD}-C$	$Fe-N_4/N_{ON}-C$	$Fe-N_4/N_{GC}-C$
*	4.92	4.92	4.92	4.92	4.92
*OOH	3.87	3.56	3.84	3.29	3.91
*O	2.03	1.92	2.06	1.58	1.96
*OH	0.74	0.39	0.69	0.14	0.77

Table S2. Calculated Gibbs free energies of ORR intermediates for $Fe-N_4-C$, $Fe-N_4/N_{BR}-C$, $Fe-N_4/N_{BD}-C$, $Fe-N_4/N_{ON}-C$, and $Fe-N_4/N_{GC}-C$ at U= 1.23 V.

	$Fe-N_4-C$	$Fe-N_4/N_{PR}-C$	$Fe-N_4/N_{PD}-C$	$Fe-N_4/N_{ON}-C$	$Fe-N_4/N_{GC}-C$
*	0	0	0	0	0
*OOH	0.18	-0.13	0.15	-0.40	0.22
*O	-0.43	-0.54	-0.40	-0.88	-0.50
*OH	-0.49	-0.84	-0.54	-1.09	-0.46

Table S3. Comparison of the raw material costs between $Fe-N_4/N_{GC}-C$, commercial Pt/C, and other previously reported catalysts. (The cost of this work is calculated based on the total material cost required to obtain the final catalyst.)

Sample	Specification	Cost (RMB g^{-1})	Retailer	References
$Fe-N_4/N_{GC}-C$	-	85	-	This work
Pt/C	20%	620	TANAKA	-
Graphdiyne	-	392,500	Energy Chemical	[7]
$HAuCl_4$	48~50%	1,088	Macklin	[8]
$IrCl_3$	≥62%	4,750		[9]
$Pd(acac)_2$	99%	1,483	Sigma-Aldrich	[10]
$RuCl_3$	99.99%	721		[11]
$RuCl_3 \cdot xH_2O$	≥99.9%	574		[12]
$Ir(acac)_3$	97%	6,622		[13]
Ti_3AlC_2 MAX phase	-	123		[14]
Graphene	electrical conductivity >10^3 S m^{-1}	5,430		[15]
$Ti_3C_2T_x$	-	11,542		[16]

Table S4. The relative contents of different N species derived from high-resolution XPS scans of N 1s.

Sample	Oxidized-N (%)	Graphitic-N (%)	Pyrrolic-N (%)	Fe-N (%)	Pyridinic-N (%)
$Fe\text{-}N_4\text{-}C$	12.26	17.22	12.26	17.86	40.40
$Fe\text{-}N_4/N_{GC}\text{-}C$	10.83	26.17	18.78	16.68	27.54

Table S5. Structural parameters of samples obtained by fitting the EXAFS data, including the average coordination number (N), path distance (R), Debye-Waller factor (σ^2), and the R-Factor of the fitting.

Sample	Path	N	R (Å)	σ^2 (Å^2)	R factor
$Fe\text{-}N_4\text{-}C$	Fe-N	4	2.00(9)	0.004(1)	0.007
$Fe\text{-}N_4/N_{GC}\text{-}C$	Fe-N	4	1.96(5)	0.008(1)	0.007

Table S6. Detailed ORR performances for catalysts in O_2-saturated 0.1 M KOH.

Catalyst	J_k @0.80 (mA cm^{-2})	J_k @0.85 (mA cm^{-2})	MA (A mg^{-1})	TOF [e/(site s)]
$Fe\text{-}N_4\text{-}C$	8.66	2.91	1.29	3.02
$Fe\text{-}N_4/N_{GC}\text{-}C$	24.76	8.13	5.61	13.15
Pt/C	21.90	6.25	0.10	0.24

Table S7. Comparison of the Cycle stability of ORR catalysts reported in recent literatures.

Catalyst	CV cycles	$E_{1/2}$ loss (mV)	Ref.
Fe_{ACs}-$VN_{NCs\ (1:1)}$/NFC	6000	16	[17]
Sb−SeNC	10,000	14	[18]
Co/SP-NC	5000	9	[19]
FeNi DSAs/N,P-PC	2000	14	[20]
FCN_4–CNN	10,000	20	[21]
Fe@MET-M	5000	10	[22]
V-CMO/5rGO	10,000	14	[23]
Pt@CoN_4-G	5000	10	[24]
FeN_4-700/900	10 000	20	[25]
Cu_{NCs}/Fe_3N-NPCF	6000	15	[26]
Fe-N-$GDCA_{0.8}$	5000	20	[27]
$NiCo_2S_4$/HCS-3	5000	8	[28]
Fe/Zn-CNHC	5000	7	[29]
FeZ-N/$S_{0.6}$-C	5000	11	[30]
Fe-FNC	5000	13	[31]
Fe-N_4/N_{GC}-C	10000	11	This work

Table S8. The fitted parameters of the EIS data of Fe-N_4/N_{GC}-C catalysts.

Potential (V)	Rs	Rct	Cdl	Rmt	Crxn
1	32.53	1834	0.0030591	51.52	0.013523
0.9	33.2	128.3	0.0042264	17.08	0.058083
0.87	33.13	113.4	0.0052196	11.08	0.05586
0.8	33.22	204.4	0.0066058	24.91	0.019859
0.7	33.09	492.2	0.0050381	36.68	0.008604
0.5	32.57	1104	0.0058187	46.85	0.011921

Table S9. The fitted parameters of the EIS data of Fe-N_4-C catalysts.

Potential (V)	Rs	Rct	Cdl	Rmt	Crxn
1	34.66	3289	0.0044919	82.2	0.0071948
0.9	32.18	294.3	0.0029614	27.94	0.0222999
0.83	32.88	148.9	0.0035519	15.83	0.014127
0.8	32.99	154.9	0.003909	26.4	0.014825
0.7	33.31	487.5	0.0039695	28.6	0.015464

0.5	34.32	1304	0.0046913	30.58	0.005064

Table S10. Comparison of the ORR performance of Fe-N_4/N_{GC}-C with recent reported Fe based catalysts.

Samples	Specific Capacity (mAh g_{Zn}^{-1})	Peak Power Density (mW cm^{-2})	References
Fe_H-N-C	814	225	[32]
Fe-N-G	802.4	133	[33]
Fe-N/C@TTAB	706.3	107.9	[34]
CoSAC@FePc	731	238.3	[35]
Fe_1/DNC+RuO_2	786.3	209.1	[36]
$NiCo_{1.8}Fe_{0.2}O_4$@NCF	802	180	[37]
FePNC	724.9	98	[38]
Fe,Co/DSA-NSC	748	240	[39]
Fe-SA@PNC	749	149	[40]
FeSA/N-PSCS	725.3	164.5	[41]
Fe-SAc/NSG	784.6	143.4	[42]
Fe-N_4/N_{GC}-C	812	225	This work

Table S11. The voltage of ZABs at different current densities.

Current density (mA cm^{-2})		1	5	10	20	50	1
Liquid ZABs	CNFs	1.25	1.21	1.19	1.15	1.04	1.23
	$Fe-N_4-C$	1.31	1.26	1.23	1.18	1.07	1.30
	$Fe-N_4/N_{GC}-C$	1.32	1.28	1.25	1.21	1.11	1.32
	Pt/C	1.33	1.29	1.26	1.22	1.12	1.28
Quasi-solid ZABs	$Fe-N_4/N_{GC}-C$	1.32	1.27	1.23	1.18	1.04	1.32
	Pt/C	1.31	1.27	1.24	1.20	1.03	1.30

References

1. Kresse, Furthmuller. Efficient iterative schemes for ab initio total-energy calculations using a plane-wave basis set. *Phys Rev B Condens* 1996; **54**: 11169-86.

2. Blochl. Projector augmented-wave method. *Phys Rev B Condens* 1994; **50**: 17953-79.

3. Perdew, Chevary, Vosko *et al.* Atoms, molecules, solids, and surfaces: Applications of the generalized gradient approximation for exchange and correlation. *Phys Rev B Condens* 1992; **46**: 6671-87.

4. Jiang R, Qiao ZL, Xu HX *et al.* Defect engineering of Fe-N-C single-atom catalysts for oxygen reduction reaction. *Chinese J Catal* 2023; **48**: 224-34.

5. Grimme S, Antony J, Ehrlich S *et al.* A consistent and accurate ab initio parametrization of density functional dispersion correction (DFT-D) for the 94 elements H-Pu. *J Chem Phys* 2010; **132**.

6. Smith DGA, Burns LA, Patkowski K *et al.* Revised damping parameters for the D3 dispersion correction to density functional theory. *J Phys Chem Lett* 2016; **7**: 2197-203.

7. Li M, Lv Q, Si W *et al.* Sp-hybridized nitrogen as new anchoring sites of Iron single atoms to boost the oxygen reduction reaction. *Angew Chem Int Ed* 2022; **61**: e202208238.

8. Yuan K, Zheng Y, Zhao Y *et al.* Bimetallic self-supported AuCu alloy aerogel with abundant diffusion channels for regulating oxygen reduction reaction by electronic structure modulation for zinc-air battery application. *Chem Eng J* 2025; **505**: 159930.

9. Yang ZY, Lai FY, Mao QJ *et al.* Breaking the mutual-constraint of bifunctional oxygen electrocatalysis via direct O—O coupling on high-valence Ir single-atom on MnO_x. *Adv Mater* 2024; **37**: 2412950.

10. Lin FX, Lv F, Zhang QH *et al.* Local coordination regulation through tuning atomic-scale cavities of Pd metallene toward efficient oxygen reduction electrocatalysis. *Adv Mater* 2022; **34**: 2202084.

11. Zhou CH, Chen X, Liu S *et al.* Superdurable bifunctional oxygen electrocatalyst for high-performance zinc-air batteries. *J Am Chem Soc* 2022; **144**: 2694-704.

12. Tamtaji M, Kim MG, Li ZM *et al.* High-throughput screening of dual atom catalysts for oxygen reduction and evolution reactions and rechargeable zinc-air battery. *Nano Energy* 2024; **126**: 109634.

13. Lv Q, Li M, Li X *et al.* Introducing hydroxyl groups to tailor the d-band center of Ir atom through side anchoring for boosted ORR and HER. *J Energy Chem* 2024; **90**: 144-51.

14. Chen SM, Liang XY, Hu SX *et al.* Inducing Fe 3*d* electron delocalization and spin-state transition of FeN_4 species boosts oxygen reduction reaction for wearable zinc-air battery. *Nano-Micro Lett* 2023; **15**: 47.

15. Ye S, Chen W, Ou Z *et al.* Harnessing the synergistic interplay between atomic-scale vacancies and ligand effect to optimize the oxygen reduction activity and tolerance performance. *Angew Chem Int Ed* 2025; **64**: e202414989.

16. Li Z, Zhuang Z, Lv F *et al.* The marriage of the FeN_4 moiety and MXene boosts oxygen reduction catalysis: Fe 3d electron delocalization matters. *Adv Mater* 2018; **30**: 1803220.

17. Wang MH, Dong Q, Ji S *et al.* "Coupling-conversion" effect induced by interface-local electric field to improve oxygen reaction kinetics in zinc-air batteries. *Chem Eng J* 2024; **481**: 148601.

18. Niu ZQ, Lu ZK, Qiao ZL *et al.* Long-range regulation of Se doping for oxygen reduction of atomically dispersed Sb catalysts for ultralow-temperature solid-state Zn-air batteries. *ACS Catal* 2023; **13**: 7122-31.

19. Chang H, Liu XY, Zhao S *et al.* Self-assembled 3D N/P/S-tridoped carbon nanoflower with highly branched carbon nanotubes as efficient bifunctional oxygen electrocatalyst toward high-performance rechargeable Zn-air Batteries. *Adv Funct Mater* 2024; **34**: 2313491.

20. Zhang L, Wu DH, Ul Haq M *et al.* Coordination engineering and electronic structure modulation of FeNi dual-single-atoms encapsulated in N, P-codoped 3D

hierarchically porous carbon electrocatalyst for synergistically boosting oxygen reduction reaction. *Appl Catal B-Environ* 2024; **351**: 123991.

21. Ryu J, Park J, Park J *et al.* Molecular engineering of atomically dispersed Fe-N_4 and Cu-N_4 dual-sites in carbon nitride nanotubes for rechargeable zinc-air batteries. *Energy Storage Mater* 2023; **55**: 397-405.

22. Li GJ, Liu JP, Xu CL *et al.* Regulating the Fe-spin state by Fe/Fe_3C neighbored single Fe-N_4 sites in defective carbon promotes the oxygen reduction activity. *Energy Storage Mater* 2023; **56**: 394-402.

23. Huang HJ, Huang AM, Liu D *et al.* Tailoring oxygen reduction reaction kinetics on perovskite oxides via oxygen vacancies for low-temperature and knittable zinc-air batteries. *Adv Mater* 2023; **35**: 2303109.

24. Zhang MT, Li H, Chen JX *et al.* A low-cost, durable bifunctional electrocatalyst containing atomic Co and Pt species for flow alkali-Al/acid hybrid fuel cell and Zn-air battery. *Adv Funct Mater* 2023; **33**: 2303189.

25. Zhao B, Xue DP, Yuan PF *et al.* Optimizing electrocatalytic oxygen reduction by adjacent C-O-C structure-driven charge separation on FeN_4 active sites. *Appl Catal B-Environ Energy* 2023; **324**: 122251.

26. Dong Q, Li GJ, Liu FF *et al.* Cu nanoclusters activating ultrafine Fe_3N nanoparticles via the Mott-Schottky effect for rechargeable zinc-air batteries. *Appl Catal B-Environ Energy* 2023; **326**: 122415.

27. Wu T, Zhu SF, Xie YM *et al.* $NaNO_3$ assisted gelatin-derived multi-level porous carbon aerogel loaded Fe single-atom for high efficient oxygen reduction reaction. *Appl Catal B-Environ Energy* 2023; **331**: 122685.

28. Liu J, Meng X, Xie JH *et al.* Dual active sites engineering on sea urchin-like CoNiS hollow nanosphere for stabilizing oxygen electrocatalysis via a template-free vulcanization strategy. *Adv Funct Mater* 2023; **33**: 2300579.

29. Li Z, Tian ZL, Cheng H *et al.* Engineering d-band center of FeN_4 moieties for efficient oxygen reduction reaction electrocatalysts. *Energy Storage Mater* 2023; **59**: 102764.

30. Zhu SF, Wu T, Liao MY *et al.* Regulating the coordination environment of

atomically dispersed Fe-N_4 moieties in carbon enables efficient oxygen reduction for Zn-air batteries. *Chem Eng J* 2024; **484**: 149693.

31. Xu CL, Guo CZ, Liu JP *et al.* Bioinspired hydrophobicity coupled with single Fe-N_4 sites promotes oxygen diffusion for efficient zinc-air batteries. *Small* 2023; **19**: 2207675.

32. Tian H, Song AL, Zhang P *et al.* High durability of Fe-N-C single-atom catalysts with carbon vacancies toward the oxygen reduction reaction in alkaline media. *Adv Mater* 2023; **35**: 2210714.

33. Gan RH, Wang YL, Zhang XW *et al.* Edge atomic Fe sites decorated porous graphitic carbon as an efficient bifunctional oxygen catalyst for Zinc-air batteries. *J Energy Chem* 2023; **83**: 602-11.

34. Niu WJ, Li RJ, Zhao WW *et al.* Hierarchical porous Fe-N/C@surfactant composites synthesized by a surfactant-assisted strategy as high-performance bifunctional oxygen electrodes for rechargeable zinc-air batteries. *J Colloid Interf Sci* 2023; **649**: 435-44.

35. Xie PF, Zhong H, Fang LZ *et al.* Molecular Fe-N_4 moieties coupled with atomic Co-N_4 sites toward improved oxygen reduction performance. *Adv Funct Mater* 2024; **34**: 2314554.

36. Ji SQ, Wang YH, Liu HX *et al.* Regulating the electronic synergy of asymmetric atomic Fe sites with adjacent defects for boosting activity and durability toward oxygen reduction. *Adv Funct Mater* 2024; **34**: 2314621.

37. Liu YY, Zhou LM, Liu SL *et al.* Fe, N-inducing interfacial electron redistribution in NiCo spinel on biomass-derived carbon for bi-functional oxygen conversion. *Angew Chem Int Ed* 2024; **63**: e202319983.

38. Liu H, Jiang LZ, Sun YY *et al.* Asymmetric N, P-coordinated single-atomic Fe sites with Fe_2P nanoclusters/nanoparticles on porous carbon nanosheets for highly efficient oxygen electroreduction. *Adv Energy Mater* 2023; **13**: 2301223.

39. Yasin G, Ali S, Ibraheem S *et al.* Simultaneously engineering the synergistic-effects and coordination-environment of dual-single-atomic iron/cobalt-sites as a bifunctional oxygen electrocatalyst for rechargeable zinc-air

batteries. *ACS Catal* 2023; **13**: 2313-25.

40. Shen MX, Qi JL, Gao K *et al.* Chemical vapor deposition strategy for inserting atomic FeN_4 sites into 3D porous honeycomb carbon aerogels as oxygen reduction reaction catalysts in high-performance Zn-air batteries. *Chem Eng J* 2023; **464**: 142719.

41. Shen MX, Liu J, Li J *et al.* Breaking the N-limitation with N-enriched porous submicron carbon spheres anchored Fe single-atom catalyst for superior oxygen reduction reaction and Zn-air batteries. *Energy Storage Mater* 2023; **59**: 102790.

42. Chi K, Wang ZP, Sun T *et al.* Simultaneously engineering the first and second coordination shells of single iron catalysts for enhanced oxygen reduction. *Small* 2024; **20**: 2311817.
